# Insights from macroevolutionary modelling and ancestral state reconstruction into the radiation and historical dietary ecology of Lemuriformes (Primates, Mammalia)

**DOI:** 10.1186/s12862-021-01793-x

**Published:** 2021-04-21

**Authors:** Ethan L. Fulwood, Shan Shan, Julia M. Winchester, Henry Kirveslahti, Robert Ravier, Shahar Kovalsky, Ingrid Daubechies, Doug M. Boyer

**Affiliations:** 1 Department of Biomedical Sciences, Kentucky College of Osteopathic Medicine, Pikeville, KY 41501 USA; 2 Department of Evolutionary Anthropology, Duke University, Durham, NC 27708 USA; 3 Department of Mathematics, Duke University, Durham, NC 27708 USA; 4 Department of Statistical Science, Duke University, Durham, NC 27708 USA; 5 Department of Electrical and Computer Engineering, Duke University, Durham, NC 27708 USA

**Keywords:** Adaptive radiation, Macroevolution, Paleobiology

## Abstract

**Background:**

Lemurs once rivalled the diversity of rest of the primate order despite thier confinement to the island of Madagascar. We test the adaptive radiation model of Malagasy lemur diversity using a novel combination of phylogenetic comparative methods and geometric methods for quantifying tooth shape.

**Results:**

We apply macroevolutionary model fitting approaches and disparity through time analysis to dental topography metrics associated with dietary adaptation, an aspect of mammalian ecology which appears to be closely related to diversification in many clades. Metrics were also reconstructed at internal nodes of the lemur tree and these reconstructions were combined to generate dietary classification probabilities at internal nodes using discriminant function analysis. We used these reconstructions to calculate rates of transition toward folivory per million-year intervals. Finally, lower second molar shape was reconstructed at internal nodes by modelling the change in shape of 3D meshes using squared change parsimony along the branches of the lemur tree. Our analyses of dental topography metrics do not recover an early burst in rates of change or a pattern of early partitioning of subclade disparity. However, rates of change in adaptations for folivory were highest during the Oligocene, an interval of possible forest expansion on the island.

**Conclusions:**

There was no clear phylogenetic signal of bursts of morphological evolution early in lemur history. Reconstruction of the molar morphologies corresponding to the ancestral nodes of the lemur tree suggest that this may have been driven by a shift toward defended plant resources, however. This suggests a response to the ecological opportunity offered by expanding forests, but not necessarily a classic adaptive radiation initiated by dispersal to Madagascar.

**Supplementary Information:**

The online version contains supplementary material available at 10.1186/s12862-021-01793-x.

## Background

The lemurs of Madagascar account for a quarter of global primate diversity, and, when recently extinct forms are considered, occupy ranges of body size, locomotor style, and dietary niche comparable to the diversity observed among all other living primates [1–3]. The taxonomic and ecological diversity of lemurs greatly exceeds that of the relatively narrowly adapted Lorisiformes, which evolved in the presence of anthropoid primates on continental Africa and Asia [4]. This diversity has prompted researchers to hypothesize that lemurs evolved on Madagascar through a process of adaptive radiation [5–10]. In this paper, predictions of an adaptive radiation model are investigated in regard to dietary adaptation using approaches that combine phylogenetic methods with the quantification of molar shape evolution.

### Adaptive radiation

The “Law of adaptive radiation” was formulated by Osborn [11] to explain the repeated evolution of mammal clades with similar ecological breadths and patterns of niche occupation on different landmasses. Simpson [12, 13] reconceptualized adaptive radiation as a process of zonal differentiation within an evolving clade as it explores a landscape of adaptive peaks previously unoccupied, or occupied by competitively inferior groups. He predicted that high rates of ecomorphological evolution will occur within groups as newly formed subclades traverse the ecological distances separating adaptive peaks. These peaks become available to evolving subclades through events that increase available ecological space. Classic examples include dispersal events, the extinction of antagonist clades, and the acquisition of “key innovations” [13–18]. Ecological opportunity may also result from the environmentally mediated opening of suitable niche space [18]. In primates, this is likely to involve the spread of tropical or paratropical forests, to which primate diversity is strongly linked [19].

### Lemur evolution

Madagascar has been separated from continental Africa since the Jurassic (~ 136 Ma), long before the plausible origins of crown strepsirrhines [20–23]. The long separation of Madagascar from Africa appears to necessitate at least one overwater dispersal coincident with the origins of Lemuriformes, likely on floating rafts of vegetation, as first proposed by Millot [24]. Modern prevailing currents transport debris discharged from African rivers north or south along the African coast and away from Madagascar, but this pattern of currents was only established during the Oligocene and early Miocene by the disruption of subtropical gyres created by the northward tectonic movements of Madagascar and Australia [21]. During most of the Paleogene, reconstructed paleocurrents would have been more amenable to dispersal from the east African coast to western Madagascar, which is reflected in the high percentage of dispersal-limited (non-volant and non-swimming) taxa that appear to have arrived on the island during this interval [22, 23]. This list of taxa includes lemurs, which are often thought to have been among the first arrivals, during the Paleocene or early Eocene [5, 22, 25, 26].

The hypothesized date for the arrival of lemurs on Madagascar is based on the estimated divergence of *Daubentonia* from the other lemurs [25]. Molecular clock estimates have returned highest probability estimates for this date between 66 and 46 Ma, encompassing the Paleocene and Eocene [25, 27–33], with more recent studies generally favoring an Eocene divergence [28, 29, 33]. Recent reexamination of the fossil taxa *Propotto* and *Plesiopithecus*, from the Miocene of Kenya and Eocene of Egypt respectively, has postulated that these taxa should be placed on the stem of *Daubentonia* (a grouping termed Chiromyiformes), complicating the traditional understanding of lemur biogeography [34]. Ancestral biogeographic reconstructions incorporating this new topology support a mainland African origin for lemurs, but two independent dispersals. This revised chronology decouples estimates of the date of the earliest dispersal of the non-chiromyiform lemurs (Lemuriformes) from the basal divergence of *Daubentonia*. Revised estimates suggest that the dispersal of Lemuriformes may have occurred as late as the Miocene, although in the absence of contradicting information, it seems likely that it must have preceded the change in current direction initiated by the northward drift of Madagascar, which likely would have occurred by the end of the Oligocene [21]. The tree topology proposed by Gunnell et al*.* [34] would also imply that the unusual ecological role of *Daubentonia* evolved while its lineage was still on the African mainland, limiting the bounds of any putative Malagasy adaptive radiation to Lemuriformes.

### Predictions of an adaptive radiation model

In an adaptive radiation model, cladogenesis is thought to be associated with the exploitation of newly accessible ecological opportunity [13]. The sweepstakes dispersal to Madagascar is often linked to the diversification of lemurs for this reason [23–25]. The tectonic history of Madagascar may have also been important in mediating lemuriform ecological opportunity, however. Madagascar reached its southern terminus during the middle Cretaceous, and has moved north on a path through the global aridity belt formed by the circulation of Hadley cells at approximately 40°–30° S latitude, likely substantially affecting climate conditions on the island [20, 21, 35]. Passage beyond the desert belt and into the influence of seasonal monsoons likely increased precipitation and drove the spread of more mesic environments, including forests, across the island during the Eocene and Oligocene [20, 22, 35]. The Eocene–Oligocene transition is also associated with widespread global cooling and drying that seems to have contributed to major extinctions of primates on northern hemisphere continents [4, 36, 37]. Comparisons between the extant mammalian faunas of Madagascar and the Paleogene faunas of mainland Africa suggest that this event may have had a winnowing effect on Madagascar as well [10]. The interaction of this global climate pattern with the local effects of tectonic movement may have created unique conditions on Madagascar spurring the diversification of lemurs during this interval.

Lemurs are sorted into speciose, apparently ecologically differentiated clades of relatively ancient origin, but most lemur species appear to have diverged only within the last few million years. Explanations of this pattern have focused on the repeated development of dispersal barriers as the landscape of Madagascar evolved across the Pleistocene [5, 38–40]. Landscape models do not address the deeper adaptive differentiation of lemur genera and families predicted by an adaptive radiation early in the history of Lemuriformes, however. Herrera [9] examined the dynamics of taxonomic and phenotypic body size diversification in lemurs using a series of phylogenetic model fitting approaches. He found evidence for accelerating rates of speciation toward the tips of the tree, supporting the suggestions of lemur diversification models that emphasize geologically recent speciation events to explain the taxonomic diversity of the extant fauna (e.g. 40). Godfrey et al. [10] revisited this diversification modelling approach, using a broader set of metrics and using the tree topologies recovered by both Kistler et al. [28] and Herrera and Dávalos [29], and found no clear evidence for either gradually increasing rates of diversification or a mass extinction followed by a rapid diversification.

The near absence of a terrestrial Tertiary fossil record on Madagascar hampers our ability to reconstruct macroevolutionary diversification dynamics across deep time on the island [5, 9]. It is particularly challenging to reconstruct the pattern of species diversification in the absence of fossil evidence [9, 41]. Adaptive radiation is expected to generate high rates of speciation early in clade history which will decline as clades diversify and fill available niches [14–16, 18, 42]. However, adaptive radiation may also involve high rates of extinction in populations morphologically intermediate between adaptive peaks, obscuring the signal of elevated speciation in the resulting branching diversification rate [42]. Many lemur species are relatively recently diverging, which may indicate iterative extinctions and diversifications of ecologically analogous taxa accompanying climate cycles across the Neogene and Quaternary, as suggested by Martin [5].

An adaptive radiation model for the origins of high-level lemur diversity would predict increased rates of ecomorphological evolution during one or both of the hypothesized periods of ecological opportunity (early Eocene or Oligocene). Phylogenetic comparative methods that fit models of evolution to phylogenies [43], or measure the accumulation of subclade disparity (the “disparity through time” method) [44], allow the detection of pulses of morphological differentiation even in clades of only extant taxa and in the context of high levels of species extinction, as reported in a study of cetacean evolution which was able to recover a signal of rapid morphological partitioning of body size even as known extinction events apparently obscured the signal of species diversification [42]. Model fitting approaches fit rate models to phylogenies given a topology and character states for tip taxa and use a maximum likelihood approach and information criteria to find the best heuristic description of the evolutionary process that produced the data [43]. Typically, in tests of adaptive radiation a Brownian motion model with a single rate constant is compared to an “early burst” model with a rate of change that declines from root to tip across a tree. The disparity through time approach was developed for clades of extant taxa and measures whether morphological divergence among extant members of clades occurs mainly at early divergences, deep in the phylogeny, consistent with an adaptive radiation accompanying cladogenesis, or among later diverging subclades toward the tips, by calculating the proportion of total clade disparity represented by subclades whose ancestors are present at each speciation event from root to tip [44].

Herrera [9] used an evolutionary modelling approach and found evidence that rates of body mass evolution showed a trended decrease after the origin of the clade, as best fit by an “early burst” model [43]. The body masses of taxa occupying different macroevolutionary niches (defined by activity pattern and diet) also showed separate evolutionary optima in a multi-peak Ornstein–Uhlenbeck model. These patterns are consistent with a partitioning into macroevolutionary niches early in the diversification of major lemur families [45]. They suggest that at least some aspects of lemur ecology may have evolved through a pattern consistent with an adaptive radiation linked to the dispersal of lemurs to Madagascar and the origins of the lemur clade.

We test predictions of an adaptive radiation model of lemur evolution using dietary ecomorphology. Although not the only aspect of lemur ecology hypothesized to have evolved by adaptive radiation [6], diet represents an important axis of high-level niche partitioning during adaptive radiation [13, 45–47]. If the high-level dietary diversity of lemurs has been shaped by a phase of general adaptive radiation, then A) High rates of adaptive evolution are expected during periods of ecological opportunity; and B) Partitioning of ecomorphological disparity is expected to occur during these intervals of ecological opportunity. We test these predictions by phylogenetically modeling the evolution of dental topography metrics, a class of tooth shape descriptors; and reconstructing the ancestral shapes of whole teeth at internal nodes of lemuriform phylogenetic trees.

## Results

### Model fitting

When considering all lemurs (lemuriformes and chiromyiformes), evolution of the dental topography metrics Dirichlet normal energy (DNE) and relief index (RFI) was best fit by a Brownian motion (BM) model of evolution (DNE: 65% of model weight; RFI: 64% of model weight). BM was still the best model when considering only groups branching after the beginning of the Oligocene (i.e. with chiromyiforms and *Megaladapis* pruned) (DNE: 49% of model weight; RFI: 62% of model weight). The coefficient of variation of DNE across the surface of the tooth (DNE CV) was best fit by an Ornstein–Uhlenbeck (OU) model (Lemurs: 91% of model weight; Oligocene lemurs: 62% of model weight). No fits preferred an early burst (EB) process predicted by an adaptive radiation (Additional file 1: Table S3).

### Disparity through time

**Fig. 1:**
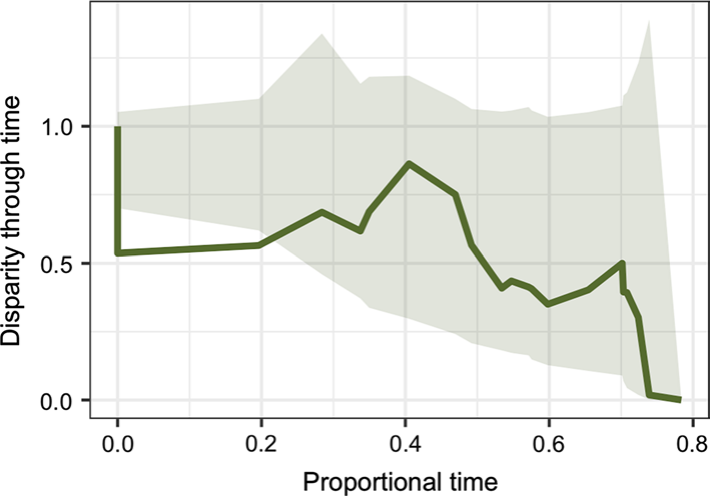
Disparity through time (DTT) plot of lemurs from the origins of the clade. Shaded region represents 95% CI of disparity modelled under a Brownian Motion process. Solid line indicates empirical disparity. Zero represents the root node of lemuriforms and lorisiforms. The calculation of DTT ends at the divergence of the most recent subclade (approximately 78% of the time distance until the present)

A significantly negative morphological disparity index (MDI) would be consistent with a lineage evolving through adaptive radiation, as this indicates the accumulation of large proportion of total clade disparity early in the divergence of major subclades [44]. MDI was negative but not significantly less than zero in the total lemuriform clade (MDI = − 0.10; p = 0.48) (Fig. 1) and positive in the clade of lemurs diverging in the Oligocene (MDI = 0.25; p = 0.95).

### Rates of evolution through time

**Fig. 2:**
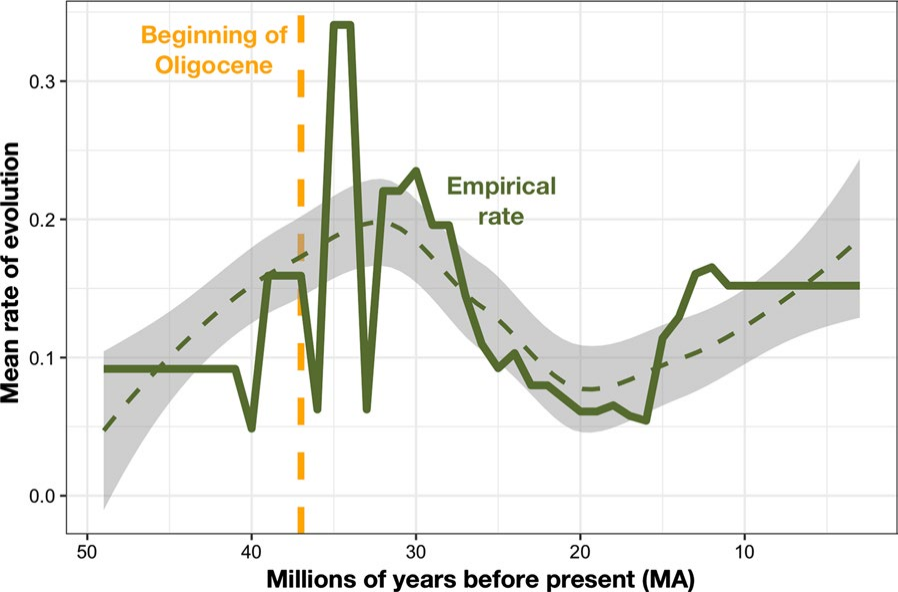
Mean rate evolution of dental adaptations for folivory at each time interval across the tree of Lemuriformes. Solid line indicates the mean rates at each bin of one-million-year duration. Dotted line with grey ribbon indicates LOWESS smoothed trend in the empirical data. The ~ 10 Ma before present shows a uniformly high mean rate of evolution, likely reflecting a “Sadler effect” of apparently rapid evolution in extant branches [47, 83]

Rates of evolution toward combinations of dental topography metrics indicative of folivory calculated using mean reconstructions (MR) visually peak during the Oligocene, coinciding with the hypothesized spread of modern Malagasy forests as the island moved into moister, tropical latitudes (Fig. 2). This is confirmed by modelling approaches. Rates of evolution were higher during the Oligocene in models using both MRs and posterior distributions of reconstructions (PDRs) (means of the posterior distribution of coefficients from the model indicating the effect of a branch crossing the Oligocene interval = 0.64; 0.19). Rates of evolution appear to be relatively low during the Eocene, contrary to the expectations of an Eocene dispersal model, which is confirmed by modeled comparisons of rates of transitions toward folivory during and after the first 10 million years of the basal divergence of lemurs (means of the posterior distribution of coefficients = − 0.18; − 0.29) (Additional file 1: Table S4; Fig. S2).

**Fig. 3:**
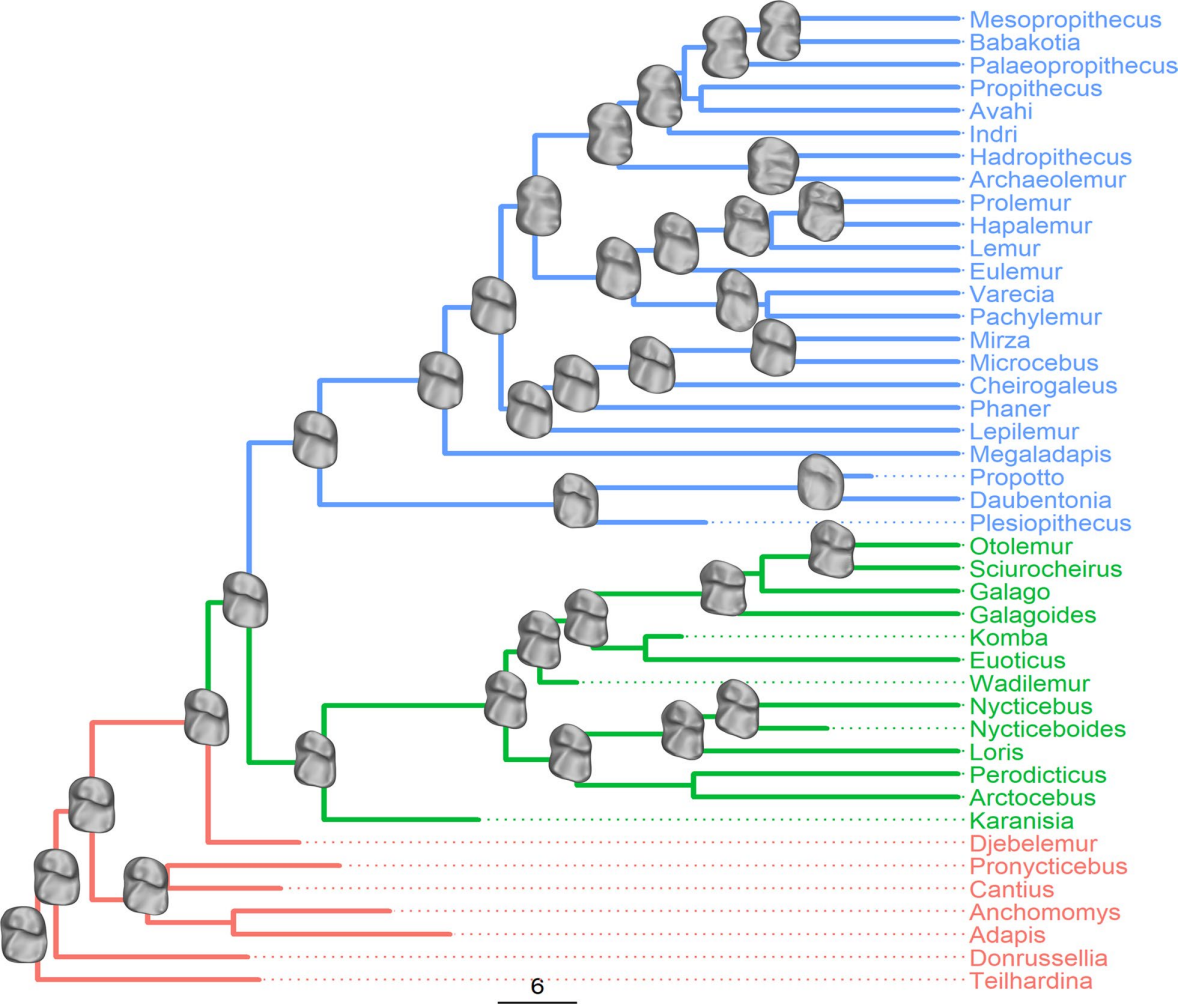
Reconstructed lower second molar morphology at internal nodes in the tree of strepsirrhines. Green branches represent lemuriforms; blue branches represent lorisiforms; Red branches represent stem strepsirrhines and other fossil primates. Scale bar indicates branch length in million years

### Ancestral tooth shapes

The morphologies of reconstructed teeth are described in detail in the Additional file 1: Figs. S2–S6. Ancestral lorisiformes and lemuriforms are broadly similar in morphology, and both resembled their last common ancestor. A major divergence occurs at the last common ancestor of the “large-bodied” lemurs, (indriidae + lemuridae), which exhibits a more bilophodont morphology (Fig. 3).

**Fig. 4:**
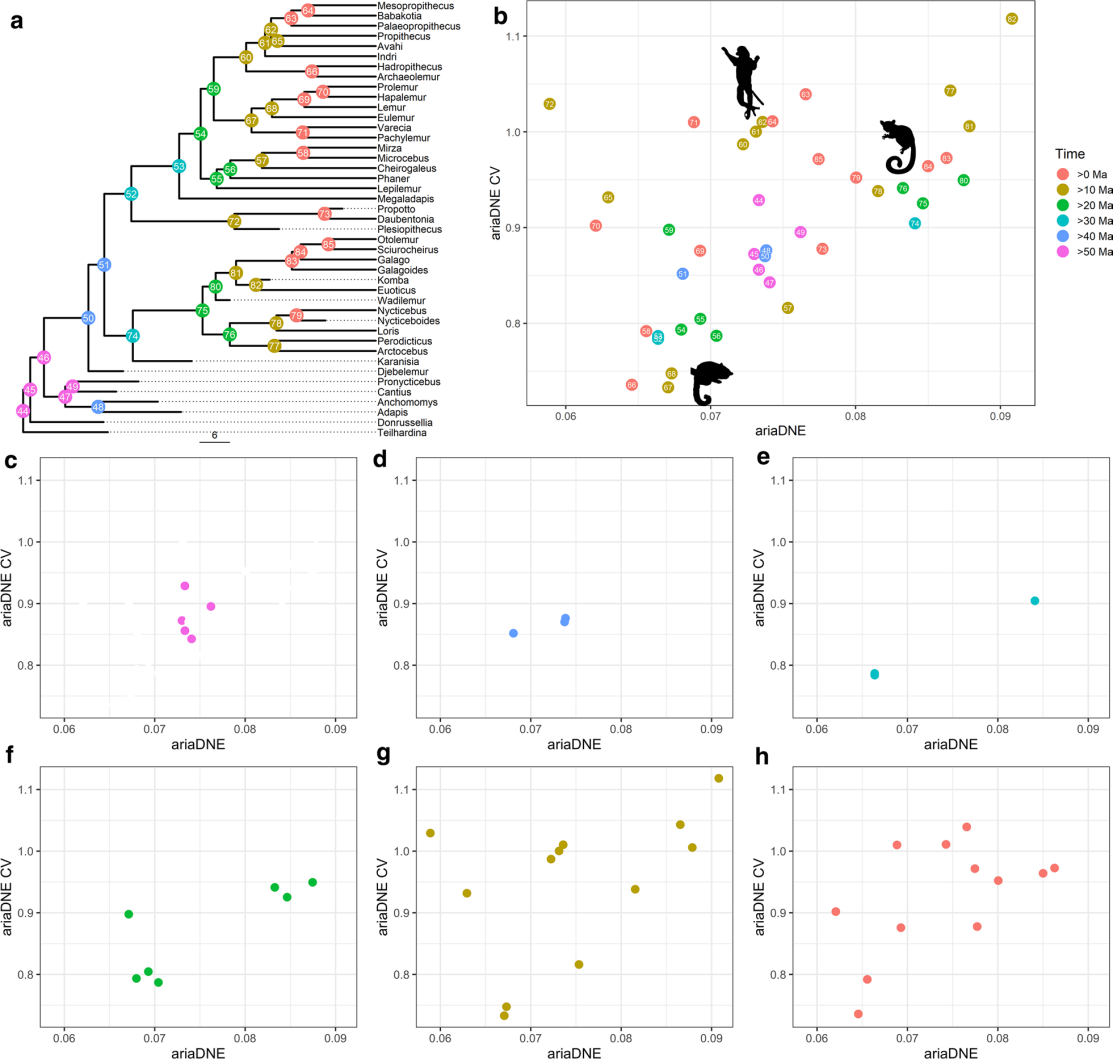
Scatterplot of DNE and DNE CV calculated on reconstructed lower second molar morphology at internal nodes in the tree of strepsirrhines, with node numbers indicated on the phylogeny. Node values are discretized into 10 million-year intervals. Scale bar indicates branch length in million years. **a** Position of nodes on phylogeny; **b** Scatterplot of nodes from all time intervals. Silhouettes represent approximate regions in which nodes of major clades cluster. Indriids are in the upper center, Lorisiformes in the upper right, and cheirogaleids in the lower left. **c**–**h** Nodes from each time interval plotted separately showing pattern of ecospace expansion

Plotting ancestral state reconstructions of DNE and DNE CV values reveals interesting patterns (Fig. 4). As might be expected under a BM model of evolution the most basal nodes cluster close together. This pattern of low variance is maintained over the first 20 million years of strepsirrhine history. From 40 to 30 Ma, ancestral lorisiform and lemuriform morphotypes strongly diverge, with lorisiforms (node 74) moving into a space of high DNE and DNE CV and lemuriforms (node 52) into a space of relatively low values for both metrics. From 30 to 20 Ma, the ancestral large-bodied (indriid + lemurid) node (node 59) diverges from those of cheirogaleids and lepilemurids. After 20 Ma, the ancestral indriids move into a mostly distinctive morphospace away from the rest of the lemurs. Lemurids expand to occupy much of the relatively low DNE region of lemur morphospace as they diversify over this interval.

## Discussion

Simpsonian adaptive radiation occurs through the “more or less simultaneous divergence of numerous lines all from much the same ancestral adaptive type into different, also diverging adaptive zones” [13]. The results of our study do not support the hypothesis that the diversity in diet of the lemuriform clade emerged as a product of Simpsonian-style adaptive radiation at the beginning of the Eocene, either as a response to dispersal to Madagascar or as a result of initial lemuriform cladogenesis. There is no signal of elevated rates of evolution in any of the three dental topography metrics, no early partitioning of morphological disparity, and relatively low rates of adaptive evolution away from the ancestral frugivorous ecology and toward folivory. Evidence for a dietary adaptive radiation of lemurs during the Oligocene is mixed. High rates of transition toward folivory are observed during the first 10 Ma of this interval, which is consistent with increased exploitation of defended plant resources in expanding and diversifying forests. However, evolutionary modelling and MDI fail to indicate rapidly divergent evolution into distinct ecological niches, at least as captured by the evolution of tooth shape descriptors.

### Model for lemur dietary evolution

**Fig. 5:**
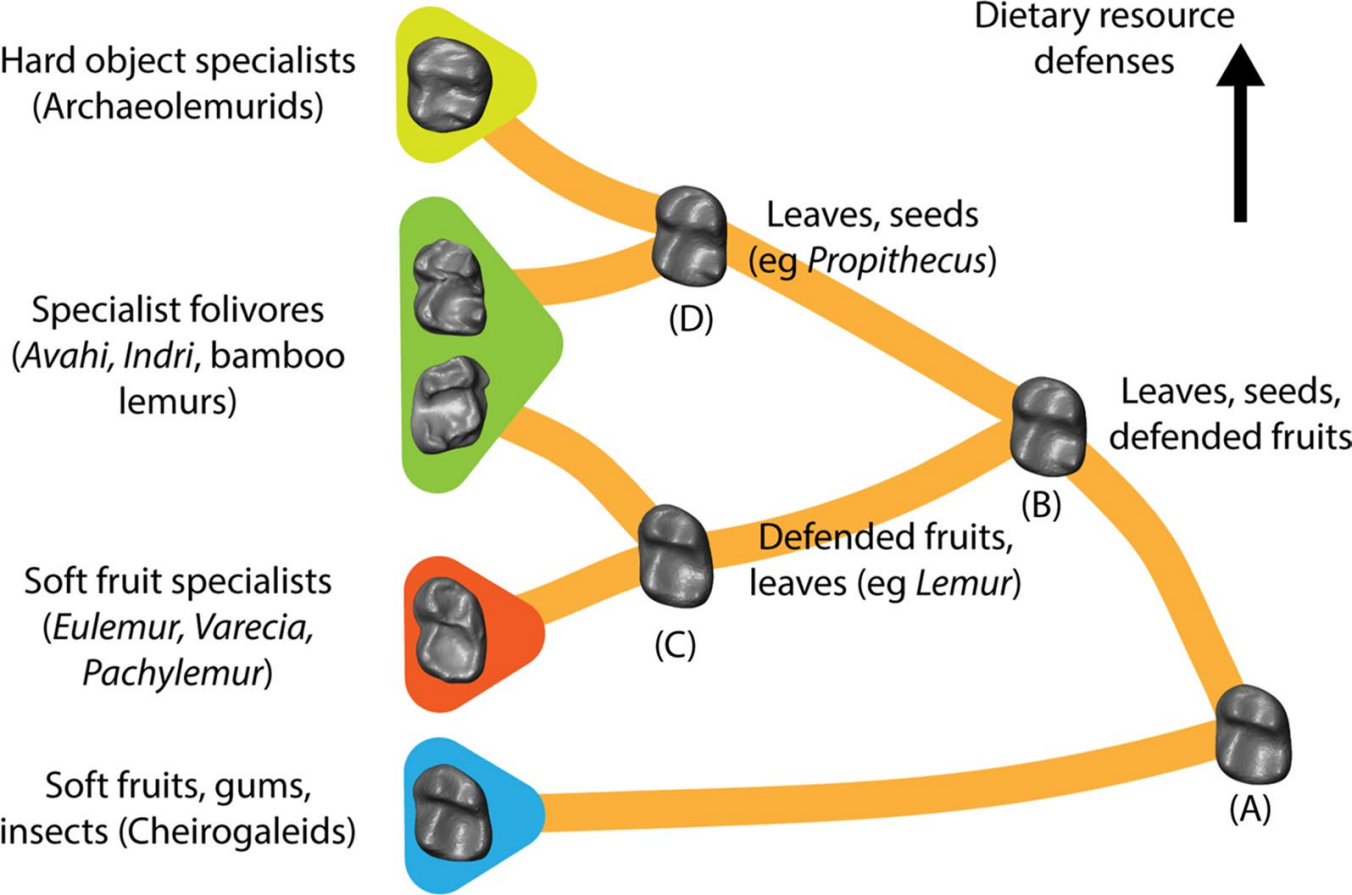
Hypothesized model for the dietary evolution of lemuriforms drawing from reconstructed lower second molar morphology and dietary ecology inferred from ancestral state reconstructions of dental topography metrics. Dietary states at internal nodes represent hypotheses for the ecologies at ancestors of extant lemur groups. Lepilemur and Megaladapis, which diverge near the origins of lemuriformes and evolve toward folivory, are not figured

Reconstructions of historical dietary ecology and molar morphology allow the tracing of a more complex model for lemur dietary evolution (Figs. 3, 5). A combination of topology of the tree and the weighted shapes reconstructed through squared change parsimony across each branch predicts the ancestral lemuriform to have resembled ancestral lorisiforms and fossil stem strepsirrhines in its molar morphology, and likely pursued a dietary ecology of mixed frugivory, gummivory, and insectivory, as supported by the reconstructions of ancestral dietary ecology using the three dental topography metrics. Cheirogaleids have continued to occupy this ecospace [48, 49]. The clade uniting lemurids and indriids (including the subfossil families Archaeolemuridae and “Palaeopropithecidae”) substantially modified the ancestral strepsirrhine molar morphology in reducing the strength of the protocristid and opening the talonid and trigonid basins. This tooth morphology approaches a more bilophodont morphology, a configuration that combines “blades” for slicing leaves with “wedges” for forcing open seeds [49, 50]. The evolution of a more bilophodont morphology at this lemurid + indriid node may indicate an important shift toward a mixed diet of fruits enclosed in hard rinds (“defended” fruits), seeds, and leaves.

At this juncture, lemurids and their sister group of indriids + archaeolemurids took divergent paths. The ancestral lemurid broadly resembles *Lemur catta* in reconstructed tooth shape, which may provide a model for the ecological origins of the group. *L. catta* consumes a mixed, seasonally shifting diet of leaves and fruits, including the well-defended pods of tamarind trees, which are enclosed in hard rinds, although this particular behavior appears to create unusual levels of tooth wear and may represent a disequilibrium in *L. catta* behavior resulting from relatively recent habitat shifts [51, 52]. From this generalist ancestor, two lemurid dietary strategies diverged. *Eulemur*, *Varecia*, and the extinct *Pachylemur* evolved toward specializations on fleshy fruits [53–55]. Adaptations for processing soft fruits include the reduction of the trigonid and expansion of the talonid basin, the reduction of the entoconid, and the incorporation of the entoconid into a continuous buccal crest without a talonid notch. *Hapalemur* and *Prolemur*, alternatively, evolved toward folivory, and in particular the exploitation of graminoid (grass-like) plants, through the enclosure of the trigonid and talonid basins in high, thickly developed crests [56, 57].

The reconstructed LCA of indriids and archaeolemurids is similar in dental morphology to the LCA of lemurids + indriids and this ancestral form may have exploited a diet broadly similar to *Propithecus*, combining fruits, seeds, and leaves in seasonally shifting combinations [55, 58–60]. Members of this clade then appear to diverge in their adaptive strategies. The LCA of archaeolemurids shares adaptations for the exploitation of hard foods with the terminal taxa *Archaeolemur* and *Hadropithecus* [61, 62]. The ancestral indriid may have shifted toward the exploitation of leaves, as indicated by the dietary ecology inferred from the reconstructed dental topography metrics at this node, while retaining adaptations for processing seeds and fruit. Molar features characteristic of indriids are present in this reconstructed ancestor, including a reduced protocristid with an expanded, open trigonid basin approaching the talonid in area, a prominent entoconid with a well-developed talonid notch, and bucco-lingually oriented crests connecting the protoconid and metaconid and entoconid and hypoconid. It is possible that these features evolved in an ancestor for which seed predation was a more important dietary activity than folivory. Highly specialized folivores like *Indri*, *Avahi*, and some of the subfossil “palaeopropithecids” arise later within this radiation [59, 63, 64].

## Conclusions

Several authors have adduced evidence for diffuse coevolutionary syndromes between lemurs and Malagasy angiosperms, suggesting an ancient and important role for the exploitation of fruits and nectar in the evolution of lemurs [65–68]. These results do not directly contradict the importance of these coevolutionary relationships among frugivorous lemurs, but do suggest that the shift toward the exploitation of defended plant resources at the origins of the lemurid + indriid clade appears to have facilitated an important expansion of these clades into morphospace unoccupied by lorisiforms and cheirogaleids (Figs. 4, 5). The acquisition of this new dietary profile occurred during the Oligocene expansion of modern Malagasy forests hypothesized to have occurred as Madagascar passed from the influence of desert latitudes into more equatorial climates [20, 35], a period that may also have coincided with the dispersal of lemuriforms to Madagascar as inferred by Gunnell et al. [34], or a recovery from a mass extinction at Eocene–Oligocene boundary driven by a global climate shock [10]. It is also possible to view the development of an incipiently bilophodont molar as a key innovation permitting the subsequent diversification of large-bodied (lemurid + indriid) lemurs. Whatever its proximate source, ecological opportunity opened by access to defended plant resources in Malagasy forests appears to have been critical in the ecological diversification of lemurs.

Our interpretation of the diversification dynamics of lemurs across deep time continues to be hampered by the lack of a Cenozoic terrestrial fossil record on Madagascar. It is possible that unrecorded radiations occurred during the evolutionary history of lemurs that generated diversity unaccounted for in models relying on the extant and subfossil faunas and included bursts of clade-wide disparity more consistent with adaptive radiation at the origins of the clade, during the Oligocene, or during some other interval [69]. This is particularly pertinent given the evidence for widespread extinctions among mammal taxa likely to have dispersed to Madagascar before the Oligocene [10]. The model presented here for the evolutionary history of lemur dietary adaptation, however, appears to capture a plausible dietary adaptive history for the extant clade in lieu of future fossil evidence. Further insight may come from the examination of other aspects of lemur ecology potentially related to the exploitation of closed forest habitats, such as locomotion. Further work is also necessary to disentangle the habitat implications of the initiation of global “icehouse” conditions at the beginning of the Oligocene and of the northward drift of Madagascar across the Eocene and Oligocene, which should have had countervailing effects on temperature and precipitation on the island. This might productively focus on the diversification dynamics and historical ecology of the flora characteristic of the different Malagasy forest zones [35].

Whether lemurs represent an exemplar of adaptive radiation relies on the framework of adaptive radiation being considered, which varies among theorists [70]. The absence of strong evidence for rapid partitioning of ecological space either at the origins of the lemuriform clade or among the lemur clades that diverge during the Oligocene may suggest a model more like that of Simpson’s “progressive occupation of numerous zones” by the evolving lemur fauna over the course of the Tertiary [13]. However, it seems quite likely that a shift toward the exploitation of defended plant foods during an Oligocene interval of ecological opportunity was critical in driving the taxonomic and ecological diversification of the extant and recently extinct lemur genera.

## Methods

### Sample

Analyses used digital meshes of second lower molars created from microCT scans of 298 specimens (Additional file 1: Table S1). This sample includes species from every extant strepsirrhine genus except for the poorly studied *Allocebus*, seven recently extinct subfossil lemurs, including species from every genus but *Archaeoindris*, and fossil primate species of the following genera: *Adapis*, *Anchomomys*, *Cantius*, *Djebelemur*, *Donrussellia*, *Karanisia*, *Komba*, *Nycticeboides*, *Plesiopithecus*, *Pronycticebus*, *Propotto*, *Teilhardina*, and *Wadilemur*. Extant taxa were classified to one of three dietary ecologies (frugivory, folivory, or insectivory) based on the largest component of their diet in field studies of wild populations (Additional file 1: Table S2). Scan processing followed procedures described in the Additional file 1.

### Tooth shape

Functional adaptation to diet is quantified using three dental topography metrics: the sum (here called DNE) and the coefficient of variation (here called DNE CV) of Dirichlet normal energy calculated across each tooth; and relief index (RFI). Dirichlet normal energy describes the curvature of the occlusal surface as deviation in normal energy from a plane [71]. DNE captures tooth sharpness, which, like shearing crest length, is associated with the processing of tough structural carbohydrates. DNE and DNE CV are calculated using the “ariaDNE” implementation, which averages vertex-by-vertex DNE across a local area determined using a bandwidth parameter [72]. Here the bandwidth parameter is set to 0.08, which indicates a local influence on each vertex calculation equivalent to 8% of the tooth surface. RFI is a ratio of a tooth’s surface area to its two-dimensional projection into an “occlusal plane” [73, 74]. Occlusal area is measured across the digitized tooth crown cropped at the enamel-cementum junction (sensu Boyer [74]). The combination of DNE, DNE CV, and RFI is effective at distinguishing folivorous from insectivorous strepsirrhines, which has previously proven challenging for tooth shape descriptors in the absence of body size information [71, 74–77]. This allows evolution of functional aspects of tooth shape to be modelled without a potentially confounding consideration of body size evolution.

### Phylogeny

Analyses used the total evidence phylogeny inferred by Herrera and Dávalos [29], which was produced by combining morphological observations on 85 extant and 33 extinct taxa and DNA from 114 extant and 8 extinct (subfossil) taxa, with fossil taxa placed into the tree using a fossilized birth–death process. The topology was modified to reflect the placement of *Plesiopithecus* and *Propotto* as sequential sister taxa to *Daubentonia* in the recent total evidence phylogeny presented by Gunnell et al*.* [34]. This phylogenetic arrangement remains controversial [10], but is likely to only minimally impact the analyses discussed below. Evolutionary model comparisons are presented with and without chiromyiform taxa included, and they were excluded in the disparity through time analysis as the ‘dtt’ function implemented in the ‘geiger’ package requires ultrametric trees [78]. Ancestral dietary reconstructions were done only on lemuriform nodes. The influence on *Plesiopithecus* and *Propotto* on the remaining analyses is confined to the reconstruction of the ancestral tooth shape and dental topography metrics at the chiromyiform + lemuriform and deeper nodes, which are not explicitly discussed here.

Taxa in the Herrera and Dávalos [29] tree for which no data were available were also pruned from the topology and species were collapsed to genera for analysis. Analysis at the genus level avoids issues arising from the reconciliation of changing understandings of lemur alpha taxonomy with data collected from museum specimens and from the uneven representation of specific diversity within genera; maximizes the sample size at each tip state; and allows questions about the differentiation of lemur morphotypes to be addressed without considering the lower-level divergence of lemur species, which may result from processes of allopatric speciation related to Quaternary climate fluctuations [40].

### Macroevolutionary model fitting

Analyses were performed on three prunings of the phylogeny: one with all lemuriform and chiromyiform taxa included, another with only the taxa diverging in the Oligocene (excluding the chiromyiformes and *Megaladapis*), and a third with all lemuriform and chiromyiform taxa but excluding the fossils *Plesiopithecus* and *Propotto* (reported in the Additional file 1). The fits of alternate models of evolution were tested for the three dental topography metrics. Adaptive radiation in lemurs, either at the origins of the clade or among the taxa diverging in the Oligocene, would be most consistent with an “early burst” (EB) model, with elevated rates of evolution early in the history of a clade [43]. EB models were tested against a null model of Brownian motion (BM) evolution and a single-optimum Ornstein–Uhlenbeck (OU) process, which models evolution using an optimum value and an attraction parameter. OU processes have been interpreted as modelling stabilizing selection or as attraction to an adaptive peak, but can also model evolutionary processes in which less phylogenetic signal is present in the distribution of a character state than would be expected by Brownian motion due to high rates of evolution toward the tips of the tree [47, 79, 80]. Model fitting was performed using the package “geiger” in R and fits were compared using the corrected Aikaike information criterion (AICc) [78, 81].

### Disparity through time

Disparity through time analysis requires an ultrametric tree, so only phylogenies excluding the fossil taxa *Plesiopithecus* and *Propotto* were considered. The two prunings were otherwise identical to those used in evolutionary model fitting. Subclade partitioning of disparity was assessed using disparity through time (DTT) analysis and the calculation of the morphological disparity index (MDI), implemented in “geiger” [44, 78]. This method calculates the relative partitioning of morphological disparity within and among subclades of a larger clade. Adaptive radiations should show relatively more morphological differentiation among early diverging subclades than within subclades, which can be visualized using a plot of the accumulation of disparity through time (DTT) from the root to the tips of a phylogeny. The deviation of the empirical accumulation of subclade disparity from the expected curve under a Brownian motion process is calculated by simulating BM evolution over 10,000 iterations. The difference in area under the empirical DTT and simulated DTT curves is calculated as the MDI. DTT curves and MDI are calculated using the squared Euclidean distances of all three dental topography metrics considered simultaneously.

### Rates of evolution through time

Rates of per-edge change in tooth shape were calculated between adjacent nodes and between nodes and adjacent tips of the lemuriform subclade. This approach allows pulses of morphological differentiation not corresponding to the origins of major clades to be identified and tested for correspondence with intervals of hypothesized ecological opportunity [82, 83].

Ancestral state reconstructions (ASR) were performed for each of the three dental topography metrics (DNE, DNE CV, and RFI) in a Bayesian framework using BayesTraits (Additional file 1). The means of the reconstructed metrics at each node were then combined in two ways to produce two outputs (here termed “mean reconstructions” = MRs and “posterior distribution of reconstructions” = PDRs). A discriminant function analysis (DFA) model was constructed, trained using extant strepsirrhines of known dietary ecology (Additional file 1: Table S2). The mean reconstructed values at each node were classified using this DFA model, generating vectors of the relative probabilities that the ancestors at each node could be classified into each of three dietary ecologies (frugivory, folivory, and insectivory) (MRs). This DFA model was also applied to a posterior distribution of 1000 reconstructions created using the complete posterior distributions of reconstructed dental topography metrics (PDRs). Estimating diet over a posterior distribution of reconstructed dental topography metrics incorporates uncertainty from both the DFA estimation of dietary ecology and from the ancestral state reconstructions of the dental topography metrics themselves. The outputs of both MR and PDR models are relative probabilities corresponding to each node and reflecting the modelled likelihood of membership in each of the three dietary ecologies by a hypothetical ancestor at that node.

Rates of change in the reconstructed probabilities that ancestral nodes represented folivores were then calculated across all of the edges in the lemur tree by taking the log difference in probabilities between adjacent nodes (or nodes and tips) and dividing by the intervening branch length, a modification of the Darwin as a unit of evolutionary rate [84–86]. Rates of evolution were calculated from both the single set of reconstructions calculated using the MRs of each node and across the 1000 iteration PDRs. Rates of change in folivory were chosen for analysis because the ancestral lemur was reconstructed as strongly frugivorous, and the relative shift toward folivory should capture the divergent acquisition of adaptations for the exploitation of defended plant resources in newly accessible or expanding forests. Calculating the rate of change in probability estimates allowed the morphological information from the three reconstructed dental topography metrics to be considered together, re-encoded in the context of biologically meaningful information on dietary ecology in strepsirrhines (in contrast to using, for example, the first principal component of a PCA).

The effects of hypothesized intervals of ecological opportunity on rates of evolution were tested using hierarchically structured Bayesian models constructed using the R package ‘brms’ (Additional file 1). Time bins of 1-million-year duration were created spanning from the initial divergence of lemurs until the present. The rates of evolution across the branches overlapping each 1 million-year time bin were assembled into vectors. Models were constructed to test whether bins occurring within the 10 Ma of the Eocene after the initial divergence of lemurs (48–38 Ma) or during the first 10 Ma of the Oligocene (34–24 Ma) show higher rates of evolution than during other intervals. This is operationally similar to ANOVA, but allows for the hierarchical clustering of rates from different BayesTraits model runs. Tests are done using only the MRs and using the PDRs aggregated by BayesTraits model run (each of the 1000 sampled iterations from the ancestral state reconstructions). Calculated rates of evolution are expected to rise artificially in branches terminating in extant taxa [47, 83]. This is comparable to the “Sadler effect” observed by stratigraphers [47, 87]. In effect, extant taxa haven’t “finished evolving,” and have shorter branches than expected by their degree of morphological differentiation. To avoid this issue, observations over the last 10 Ma are excluded.

### Ancestral shape reconstruction

Shape evolution is examined using a novel application of ancestral state reconstruction to continuous, landmark-free representations of 3D shape to generate digital mesh objects representing the ancestral morphologies characterizing nodes on the phylogenetic tree of strepsirrhines, focusing on extant and recently extinct subfossil lemurs. Mesh files representing the second lower molars of tip taxa are first aligned using Auto3dgm and then registered using a preliminary version of SAMS, an open-source software suite improving on continuous Procrustes methods [88–92]. Ancestral shapes are computed using the weighted means of the positions of each digital model vertex using squared changed parsimony (equivalent to a Brownian Motion model of evolution in a maximum likelihood framework [93]) across branches of the tree, a modification of approaches to calculating mean shape using the positions of a set of homologous landmarks (94). Models fit to the evolution of DNE and RFI best supported a BM process, suggesting that this may accurately approximate the evolution of tooth shape. Future refinements of this method for reconstructing mean shapes will explore methods for fitting more complex evolutionary models. DNE and DNE CV are then calculated from reconstructed meshes to examine trends in dietary adaptation at internal nodes.

## Supplementary Information

**Additional file 1.** Methodological details; additional results figures and tables; descriptions of reconstructed ancestral dental morphologies; DOI of lower molar scans, available on Morphosource.

## Data Availability

Digital models of the teeth analyzed in this article are available on the Morphosource database Morphosource, with doi provided in the additional material.

## Abbreviations

DNE: Dirichlet normal energy
DNE CV: Coefficient of variation of the Dirichlet normal energy
RFI: Relief index
BM: Brownian motion
OU: Ornstein–Uhlenbeck
EB: Early burst
DTT: Disparity through time
MDI: Morphological disparity index
ASR: Ancestral state reconstruction
LCA: Last common ancestor
PCA: Principal component analysis
DFA: Discriminant function analysis
MNHN: Museum of Natural History, Paris
NHMB: Museum of Natural History, Basel
NMB: Natural History Museum, Bern
YPM: Yale Peabody Museum of Natural History
CGM: Cairo Geological Museum
DPC: Division of Fossil Primates, Duke Lemur Center
USNM: Smithsonian National Museum of Natural History
AMNH: American Museum of Natural History
BMNH: Natural History Museum, London
MCZ: Museum of Comparative Zoology, Harvard University
UM: University of Michigan
FMNH: Field Museum of Natural History
SBU: Stony Brook University
CBI: National Office of Mines, Tunisia
UWBM: Burke Museum, University of Washington
NHMW: Museum of Natural History, Vienna
KNM: National Museums of Kenya
UAAC: Museum of Zoology, University of Calgary
UA: University of Antananarivo
YGSP: Geological Survey of Pakistan
UAMZ: Museum of Zoology, University of Alberta
IRSNB: Royal Belgian Institute of Natural Sciences
