# Supplementary material for: Insights from macroevolutionary modelling and ancestral state reconstruction into the radiation and historical dietary ecology of Lemuriformes (Primates, Mammalia)
Source: BMC Ecol Evol. 2021 Apr 21;21:60. doi: 10.1186/s12862-021-01793-x (PMC8061064; doi:10.1186/s12862-021-01793-x)
Supplement: Supplementary file 1 — Additional file 1. Methodological details; additional results figures and tables; descriptions of reconstructed ancestral dental morphologies; DOI of lower molar scans, available on Morphosource. [file 12862_2021_1793_MOESM1_ESM.docx]

# Additional methods

### Scan Processing

Scans were performed on osteological and fossil material and on casts created from polyvinylsiloxane molds. Mesh “.ply” files were created by segmenting microCT tiff stacks in Avizo (version 8; Visualization Sciences Group, Burlington, Mass., USA). Surfaced objects, which were typically entire mandibles with several tooth positions preserved, were smoothed over 20 iterations and the second lower molar was cropped from each scan using Geomagic (3D Systems, Rock Hill, SC). Each molar was then simplified to 10,000 faces and smoothed again over 20 iterations. Smoothing was kept to 20 iterations at each step to avoid the introduction of mesh irregularities at higher numbers of iterations [1].

### Analysis steps

Ancestral state reconstructions were performed in BayesTraits V3, accessed through the BayesTraits wrapper pacakge “btw” in R. Reconstructions were performed in a Bayesian framework using the program BayesTraits over 1,025,000 iterations with a 25,000 iteration burnin and sampling at every 1,000 iterations. Model testing was performed using a stepping stone sampler and Bayes Factors. These tests preferred random walk to directional models for all three metrics. Because model fitting strongly preferred an OU process for the evolution of DNE CV, an OU tree-stretching metric was incorporated into the Bayesian reconstruction of DNE CV, the value of which was jointly modelling with the ancestral states. Bayesian ancestral state reconstruction generated a posterior distribution of 1000 reconstructions at each node.

Additional analyses used the package “MASS” (for discriminant function analysis) and custom functions. Bayesian hierarchical model fitting was performed using the package “brms” [2]. Four chains were run in brms over 3000 iterations, with a 1000 iteration warmup. Chain convergence was assessed using the rhat parameter, the number of effective samples returned, and visual inspection of the chain trace plots.

# Supplemental Results


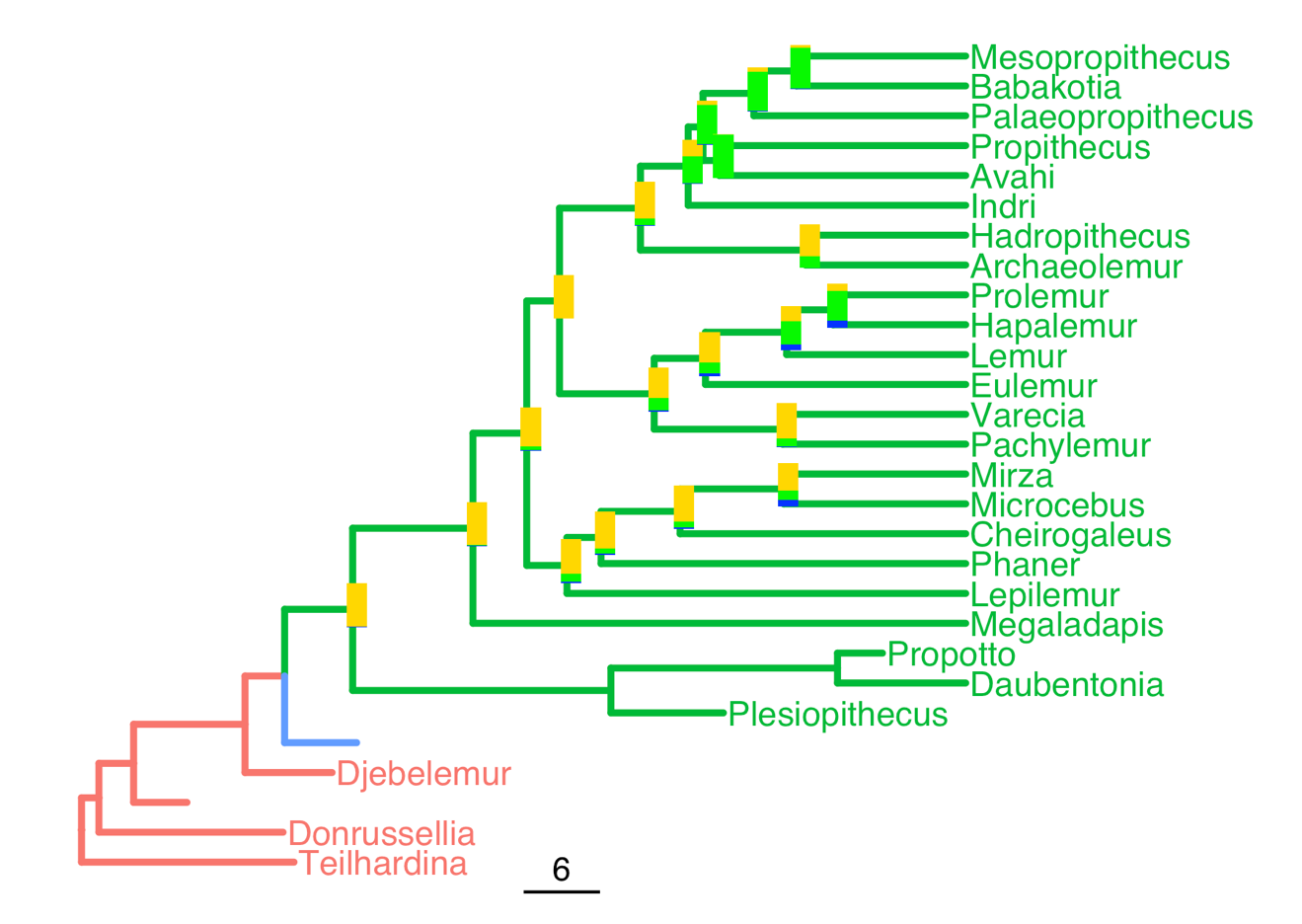


Figure S1: Ancestral dietary ecologies reconstructed at internal nodes of the lemur tree from combinations of the mean reconstructed dental topography metrics DNE, DNE CV, and RFI. At nodes, the proportions of each bar indicate the relative reconstructed each dietary ecology. Yellow = Frugivory, Green = Folivory, Blue = Insectivory. Green branches represent lemuriforms; blue branch is a pruned lorisiform branch; Red branches represent stem strepsirrhines and other fossil euprimates.

### Descriptions of reconstructed ancestral tooth shapes

Dental terminology follows Szalay and Delson [3]. Lorisiforms retain from stem strepsirrhines a relatively conservative molar morphology, with a mesiodistal narrowing of the trigonid basin representing the most prominent divergent trend (Figure 1). Among ancestral lorisiform nodes, including the LCA of Lorisiformes, of crown Lorisiformes, of galagids, and of lorisids, primitive retentions include a strong cristid obliqua which comes close to the midline of a strong protocristid, a relatively buccolingually closed talonid basin, and an entoconid connected to the protocristid by a strong pre-entocristid but with a weak postcristid.


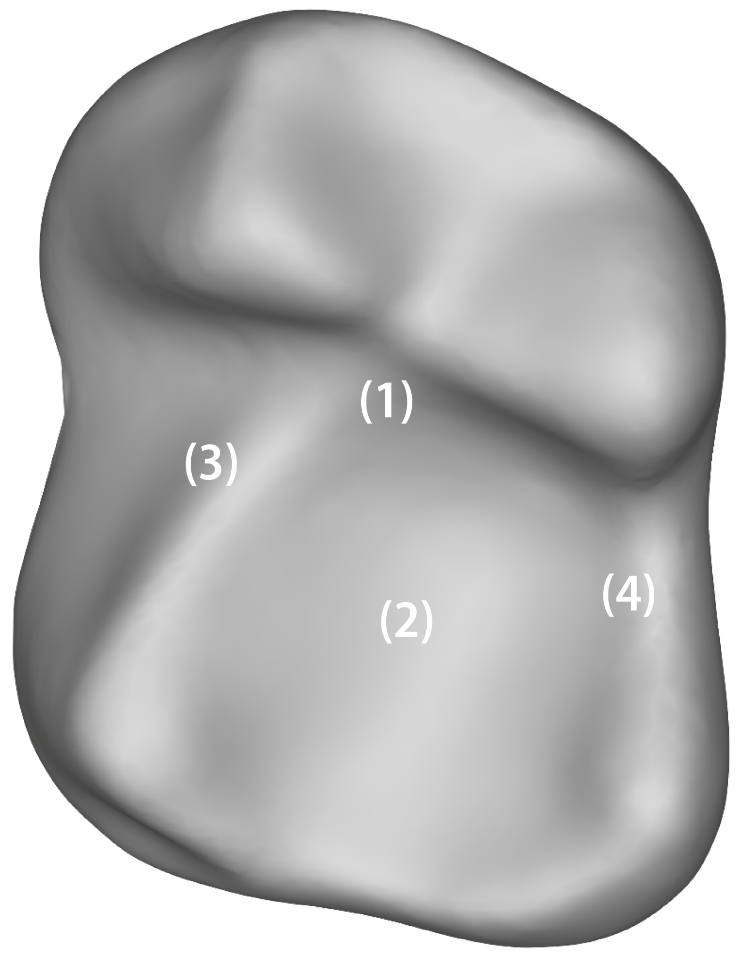


Figure S2: Reconstructed ancestral lorisiform lower second molar morphology. 1) Strong protocristid; 2) Closed talonid basin; 3) Strong cristid obliqua; 4) Continuous pre-entocristid

The reconstructed ancestral lemuriform molar shares much of this morphology, but with slightly expanded talonid and trigond basins and reduced “waisting” in the region of the crstid obliqua (Figure 2). This morphology is also reconstructed for the LCAs of Lemuriformes + Chiromyformes, the cheirogaleid + lepilemurid LCA, and the ancestral cheirogaleid. *Lepilemur* and *Megaladapis* appear to derive their unusual dental morphologies in parallel from this generalized strepsirrhine dental morphology along long branches. These lineages may have been the first to shift toward the exploitation of defended plant resources (primarily leaves) on Madagascar.


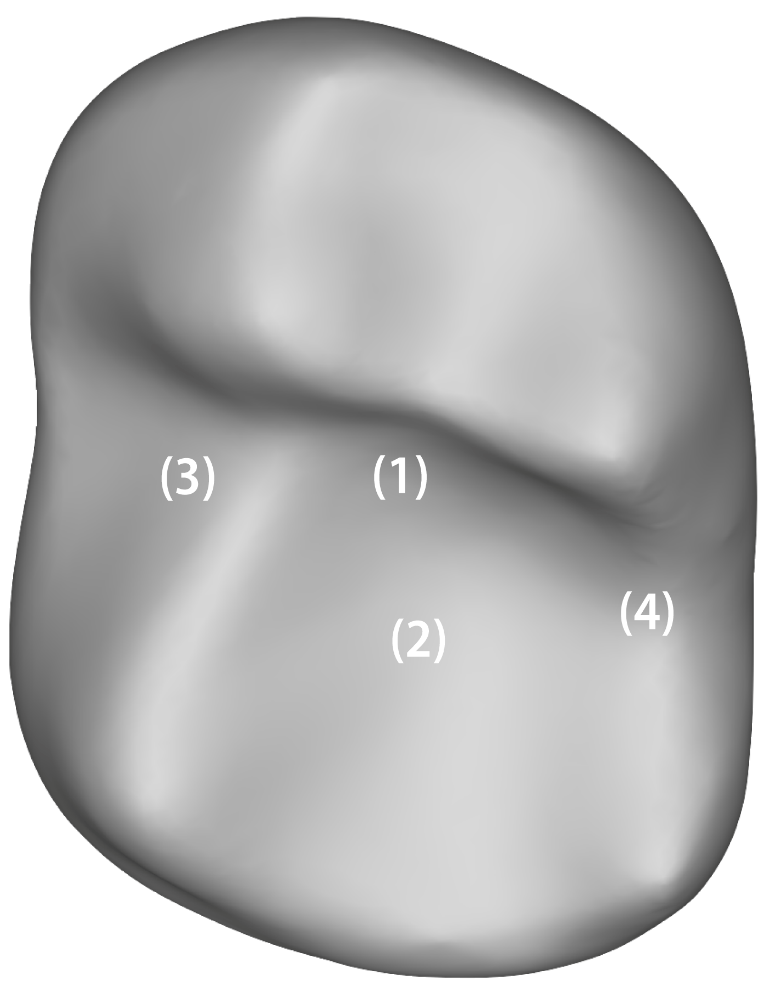


Figure S3: Reconstructed ancestral lemuriform lower second molar morphology. 1) Strong protocristid; 2) Expanded talonid basin; 3) Strong cristid obliqua, with reduced waisting; 4) Continuous pre-entocristid

The LCA of the indriid + lemurid clade is sister to the cheirogaleid + lepilemurid clade. Interestingly, reconstructed molar morphology at this node more closely resembles extant indriids than it does the ancestral lemuriform or extant lemurids (Figure 3). Indriid features include an open trigonid basin with a rounded paralophid; weakly developed protocristid; open talonid basin with a cristid obliqua meeting the protocristid buccally; strongly developed metaconid and entoconid; and entoconid isolated from the metaconid by a deep talonid notch. The LCAs of indriids + archaeolemurids and of Indriidae all share these traits, developed to a greater degree as the nodes approach the tips within Indriidae (Figure 4). The ancestor of the “paleopropithecids” (nested in this tree within Indriidae) also shares this profile. Later nodes show a stronger development of buccolingual crests linking the metaconid and entoconid with the protoconid and hypoconid, a characteristic feature of the bilophodont morphology of extant indriids.


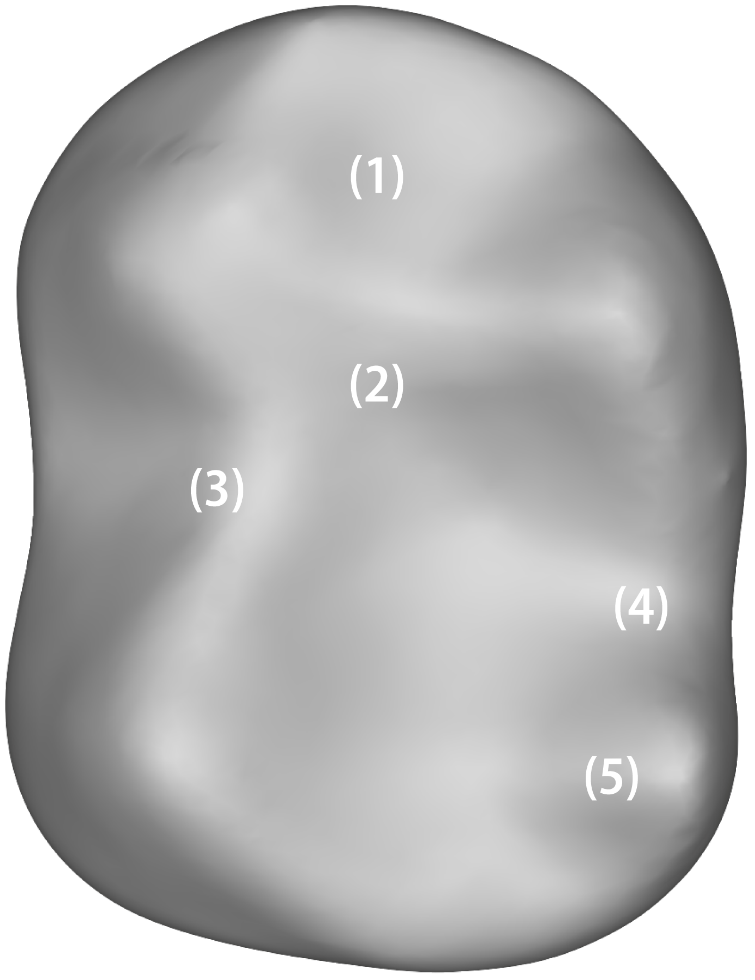


Figure S4: Reconstructed ancestral indriid + lemurid lower second molar morphology. 1) Expanded trigonid with keel-shaped paralophid; 2) Weak protocristid; 3) Buccally shifted cristid obliqua; 4) Talonid notch; 5) Prominent entoconid.


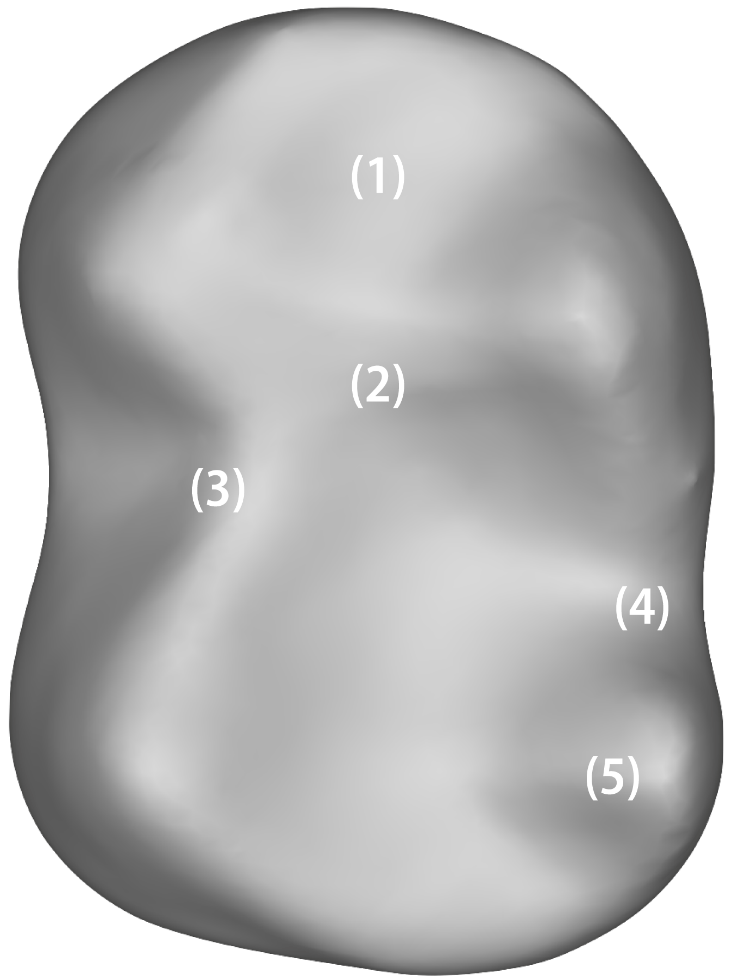


Figure S5: Reconstructed ancestral indriid + archaeolemurid lower second molar morphology. 1) Expanded trigonid with keel-shaped paralophid; 2) Weak protocristid; 3) Buccally shifted cristid obliqua; 4) Talonid notch; 5) Prominent entoconid.

The reconstructed ancestral lemurid morphology indicates a partial reversal of this morphological trend (Figure 5). Molars reconstructed at internal lemurid nodes show stronger protocristids, a strongly developed paralophid enclosing an oval-shaped trigonid basin, and an entoconid isolated from the metaconid by a talonid notch. This configuration broadly resembles the morphology of *Lemur catta*. The shape and position of the entoconid appears to have reversed from this condition in extant *Eulemur* and in the node linking *Varecia* and *Pachylemur*, where it is weakly developed and enclosed in a continuous, strong pre-entocristid and postcristid.


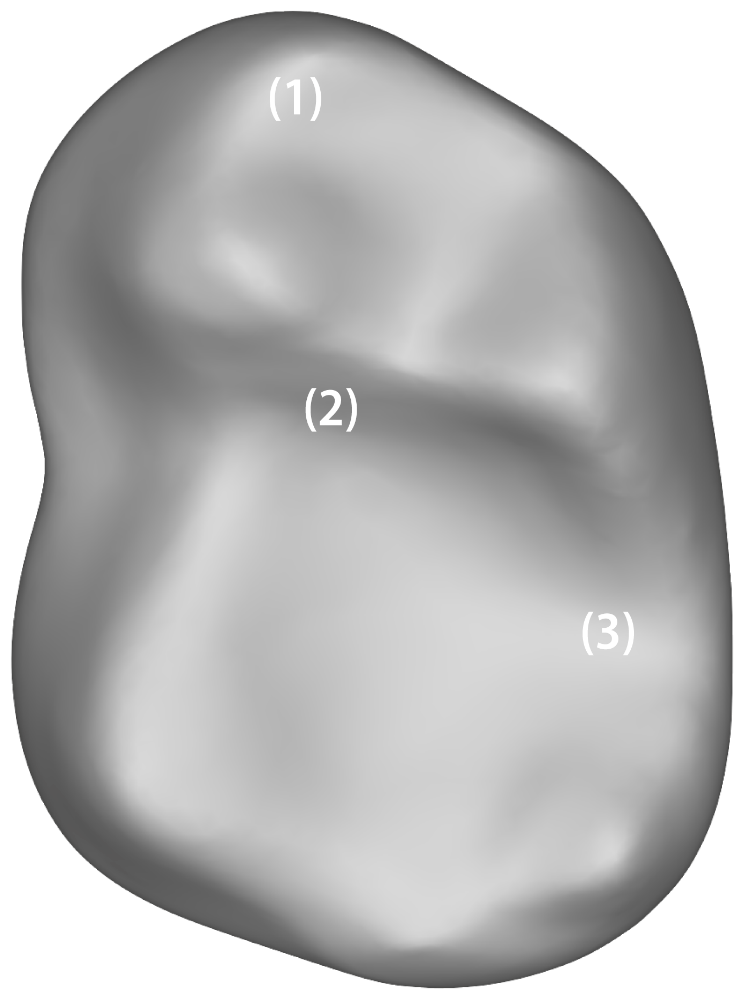


Figure 6: Reconstructed ancestral lemurid lower second molar morphology. 1) Strong paralophid; 2) Strong protocristid; 3) Talonid notch

# Additional tables

Table S1: Specimens analyzed

| **Genus** | **Species** | **Collection** | **Specimen** | **Resolvable, unique permanent data ID** |
| --- | --- | --- | --- | --- |
| *Adapis* | *parisiensis* | MNHN-Qu | 10966 | https://doi.org/10.17602/M2/M46638 |
| *Adapis* | *parisiensis* | MNHN-Qu | 11032 | https://doi.org/10.17602/M2/M46506 |
| *Adapis* | *parisiensis* | MNHN-Qu | 11046 | https://doi.org/10.17602/M2/M46519 |
| *Adapis* | *parisiensis* | MNHN-Qu | 11069 | https://doi.org/10.17602/M2/M46540 |
| *Adapis* | *parisiensis* | MNHN-Qu | 11117 | https://doi.org/10.17602/M2/M46647 |
| *Adapis* | *parisiensis* | MNHN-Qu | 11166 | https://doi.org/10.17602/M2/M46551 |
| *Adapis* | *parisiensis* | MNHN-Qu | 20940 | https://doi.org/10.17602/M2/M46591 |
| *Adapis* | *parisiensis* | NHMB-QW | 9 | https://doi.org/10.17602/M2/M46634 |
| *Adapis* | *parisiensis* | NMB-Ql | 71 | https://doi.org/10.17602/M2/M46643 |
| *Adapis* | *parisiensis* | YPM-VP | 30440 | https://doi.org/10.17602/M2/M46661 |
| *Anchomomys* | *frontayensis* | CGM | 42842 | https://doi.org/10.17602/M2/M56814 |
| *Archaeolemur* | *majori* | MNHN | 1935-416 | https://doi.org/10.17602/M2/M82637 |
| *Archaeolemur* | *sp* | DPC | 10858 | https://doi.org/10.17602/M2/M82638 |
| *Archaeolemur* | *sp* | DPC | 10899 | https://doi.org/10.17602/M2/M82639 |
| *Archaeolemur* | *sp* | DPC | 11085 | https://n2t.net/ark:/87602/m4/M53794 |
| *Archaeolemur* | *sp* | DPC | 11728 | https://n2t.net/ark:/87602/m4/M82640 |
| *Arctocebus* | *calabarensis* | USNM | 377275 | https://n2t.net/ark:/87602/m4/M619 |
| *Arctocebus* | *calabariensis* | AMNH | 207949 | https://doi.org/10.17602/M2/M53225 |
| *Arctocebus* | *calabariensis* | AMNH | 297949 | https://doi.org/10.17602/M2/M46669 |
| *Arctocebus* | *calabariensis* | BMNH | 48.324 | https://doi.org/10.17602/M2/M46700 |
| *Arctocebus* | *calabariensis* | BMNH | 48.582 | https://doi.org/10.17602/M2/M46736 |
| *Arctocebus* | *calabariensis* | BMNH | 48.586 | https://doi.org/10.17602/M2/M46747 |
| *Arctocebus* | *calabariensis* | USNM | 395710 | https://doi.org/10.17602/M2/M46989 |
| *Arctocebus* | *calabariensis* | USNM | 511930 | https://n2t.net/ark:/87602/m4/M623 |
| *Avahi* | *laniger* | AMNH | 41267 | https://doi.org/10.17602/M2/M42 |
| *Avahi* | *laniger* | AMNH | 100635 | https://n2t.net/ark:/87602/m4/M54 |
| *Avahi* | *laniger* | AMNH | 170451 | https://doi.org/10.17602/M2/M56609 |
| *Avahi* | *laniger* | AMNH | 170461 | https://doi.org/10.17602/M2/M46 |
| *Avahi* | *laniger* | AMNH | 170501 | https://doi.org/10.17602/M2/M26 |
| *Avahi* | *laniger* | MCZ | 32503 | https://doi.org/10.17602/M2/M56738 |
| *Avahi* | *laniger* | MCZ | 44878 | https://doi.org/10.17602/M2/M56741 |
| *Avahi* | *laniger* | MCZ | 44879 | https://doi.org/10.17602/M2/M56742 |
| *Avahi* | *laniger* | USNM | 83650 | https://doi.org/10.17602/M2/M17 |
| *Avahi* | *laniger* | USNM | 83652 | https://doi.org/10.17602/M2/M23 |
| *Avahi* | *sp* | DPC | 12818 | https://n2t.net/ark:/87602/M4/M29584 |
| *Babakotia* | *sp* | DPC | 10970 | https://n2t.net/ark:/87602/M4/M29579 |
| *Babakotia* | *sp* | DPC | 11799 | https://n2t.net/ark:/87602/M4/M29580 |
| *Babakotia* | *sp* | DPC | 11800 | https://n2t.net/ark:/87602/M4/M29581 |
| *Babakotia* | *sp* | DPC | 11801 | https://n2t.net/ark:/87602/M4/M29851 |
| *Babakotia* | *sp* | DPC | 12772 | https://n2t.net/ark:/87602/M4/M29583 |
| *Cantius* | *mckennai* | UM | 76250 | https://doi.org/10.17602/M2/M47351 |
| *Cantius* | *mckennai* | UM | 76394 | https://doi.org/10.17602/M2/M47355 |
| *Cantius* | *mckennai* | UM | 78929 | https://doi.org/10.17602/M2/M47680 |
| *Cantius* | *ralstoni* | UM | 65133 | https://doi.org/10.17602/M2/M47222 |
| *Cantius* | *ralstoni* | UM | 75358 | https://doi.org/10.17602/M2/M47347 |
| *Cantius* | *torresi* | CAB | 04-274 | https://doi.org/10.17602/M2/M47692 |
| *Cantius* | *torresi* | UM | 87852 | https://doi.org/10.17602/M2/M47687 |
| *Cantius* | *torresi* | UM | 101958 | https://doi.org/10.17602/M2/M47684 |
| *Cantius* | *trigonodus* | UM | 85950 | https://doi.org/10.17602/M2/M47676 |
| *Cheirogaleus* | *major* | AMNH | 31265 | https://doi.org/10.17602/M2/M55772 |
| *Cheirogaleus* | *major* | AMNH | 80072 | https://doi.org/10.17602/M2/M130 |
| *Cheirogaleus* | *major* | AMNH | 100640 | https://doi.org/10.17602/M2/M58 |
| *Cheirogaleus* | *major* | AMNH | 100830 | https://doi.org/10.17602/M2/M66 |
| *Cheirogaleus* | *medius* | AMNH | 100654 | https://doi.org/10.17602/M2/M143 |
| *Cheirogaleus* | *medius* | AMNH | 196618 | https://doi.org/10.17602/M2/M147 |
| *Cheirogaleus* | *medius* | DPC | 34 | https://n2t.net/ark:/87602/M4/M24206 |
| *Cheirogaleus* | *medius* | DPC | 138 | https://n2t.net/ark:/87602/M4/M24214 |
| *Cheirogaleus* | *major* | FMNH | 5656 | https://doi.org/10.17602/M2/M47757 |
| *Cheirogaleus* | *medius* | FMNH | 85146 | https://doi.org/10.17602/M2/M47759 |
| *Cheirogaleus* | *medius* | FMNH | 147986 | https://doi.org/10.17602/M2/M47760 |
| *Daubentonia* | *madagascariensis* | AMNH | 41334 | https://doi.org/10.17602/M2/M110 |
| *Daubentonia* | *madagascariensis* | AMNH | 100632 | https://doi.org/10.17602/M2/M102 |
| *Daubentonia* | *madagascariensis* | SBU | no number | https://doi.org/10.17602/M2/M469 |
| *Djebelemur* | *sp* | CBI | 366 | https://doi.org/10.17602/M2/M82659 |
| *Donrussellia* | *gallica* | MNHN | 4854 | https://doi.org/10.17602/M2/M47833 |
| *Donrussellia* | *gallica* | MNHN | 7655 | https://doi.org/10.17602/M2/M47817 |
| *Donrussellia* | *provincialis* | MNHN | 170 | https://doi.org/10.17602/M2/M47831 |
| *Donrussellia* | *provincialis* | MNHN | 4598 | https://doi.org/10.17602/M2/M47821 |
| *Eulemur* | *fulvus* | AMNH | 18696 | https://doi.org/10.17602/M2/M126 |
| *Eulemur* | *fulvus* | AMNH | 19159 | https://doi.org/10.17602/M2/M122 |
| *Eulemur* | *fulvus* | AMNH | 100517 | https://doi.org/10.17602/M2/M48026 |
| *Eulemur* | *fulvus* | DPC | 6696 | https://n2t.net/ark:/87602/M4/M29569 |
| *Eulemur* | *fulvus* | USNM | 63338 | https://doi.org/10.17602/M2/M15 |
| *Eulemur* | *fulvus albifrons* | AMNH | 100559 | https://doi.org/10.17602/M2/M55775 |
| *Eulemur* | *fulvus albifrons* | AMNH | 100586 | https://doi.org/10.17602/M2/M55776 |
| *Eulemur* | *macaco* | UWBM | 39000 | https://doi.org/10.17602/M2/M48078 |
| *Eulemur* | *rufus* | AMNH | 41264 | https://doi.org/10.17602/M2/M47992 |
| *Eulemur* | *rufus* | AMNH | 41268 | https://doi.org/10.17602/M2/M171 |
| *Eulemur* | *rufus* | AMNH | 100569 | https://doi.org/10.17602/M2/M159 |
| *Eulemur* | *rufus* | USNM | 83961 | https://doi.org/10.17602/M2/M156 |
| *Eulemur* | *sp* | DPC | 3750 | https://n2t.net/ark:/87602/M4/M29569 |
| *Eulemur* | *sp* | FMNH | 171090 | https://doi.org/10.17602/M2/M82662 |
| *Euoticus* | *elegantulus* | AMNH | 269928 | https://doi.org/10.17602/M2/M56632 |
| *Euoticus* | *elegantulus* | MCZ | 14657 | https://doi.org/10.17602/M2/M56730 |
| *Euoticus* | *elegantulus* | MCZ | 17590 | https://doi.org/10.17602/M2/M56733 |
| *Euoticus* | *elegantulus* | MCZ | 17591 | https://doi.org/10.17602/M2/M56734 |
| *Euoticus* | *elegantulus* | MCZ | 17593 | https://doi.org/10.17602/M2/M56735 |
| *Euoticus* | *elegantulus* | MCZ | 18609 | https://doi.org/10.17602/M2/M56736 |
| *Euoticus* | *elegantulus* | USNM | 598465 | https://doi.org/10.17602/M2/M48111 |
| *Sciurocheirus* | *alleni* | AMNH | 236349 | https://doi.org/10.17602/M2/M175 |
| *Sciurocheirus* | *alleni* | MCZ | 14659 | https://doi.org/10.17602/M2/M56731 |
| *Sciurocheirus* | *alleni* | MCZ | 17589 | https://doi.org/10.17602/M2/M56732 |
| *Sciurocheirus* | *alleni* | MCZ | 19969 | https://doi.org/10.17602/M2/M56737 |
| *Sciurocheirus* | *alleni* | MCZ | 64170 | https://doi.org/10.17602/M2/M56746 |
| *Sciurocheirus* | *alleni* | USNM | 84534 | https://doi.org/10.17602/M2/M48151 |
| *Galagoides* | *demidovii* | AMNH | 50984 | https://doi.org/10.17602/M2/M48151 |
| *Galagoides* | *demidovii* | AMNH | 104804 | https://doi.org/10.17602/M2/M55771 |
| *Galagoides* | *demidovii* | AMNH | 119810 | https://doi.org/10.17602/M2/M183 |
| *Galagoides* | *demidovii* | AMNH | 239438 | https://doi.org/10.17602/M2/M191 |
| *Galagoides* | *demidovii* | AMNH | 241122 | https://doi.org/10.17602/M2/M195 |
| *Galagoides* | *demidovii* | AMNH | 241124 | https://doi.org/10.17602/M2/M187 |
| *Galagoides* | *demidovii* | SBU | Ga-04 | https://doi.org/10.17602/M2/M53182 |
| *Galagoides* | *demidovii* | USNM | 598467 | https://doi.org/10.17602/M2/M53194 |
| *Galago* | *senegalensis* | AMNH | 187359 | https://doi.org/10.17602/M2/M199 |
| *Galago* | *senegalensis* | AMNH | 187360 | https://doi.org/10.17602/M2/M203 |
| *Galago* | *senegalensis* | AMNH | 187362 | https://doi.org/10.17602/M2/M207 |
| *Galago* | *senegalensis* | FMNH | 186919 | https://doi.org/10.17602/M2/M53154 |
| *Galago* | *senegalensis* | MCZ | 34381 | https://doi.org/10.17602/M2/M56739 |
| *Hadropithecus* | *stenognathus* | NHMW | 1934.IV.2/1a | https://doi.org/10.17602/M2/M82663 |
| *Hapalemur* | *griseus* | AMNH | 100628 | https://doi.org/10.17602/M2/M55840 |
| *Hapalemur* | *griseus* | AMNH | 170672 | https://doi.org/10.17602/M2/M56621 |
| *Hapalemur* | *griseus* | MCZ | 44921 | https://doi.org/10.17602/M2/M56743 |
| *Hapalemur* | *griseus* | USNM | 83668 | https://n2t.net/ark:/87602/m4/M607 |
| *Hapalemur* | *griseus* | USNM | 84386 | https://n2t.net/ark:/87602/m4/M611 |
| *Hapalemur* | *griseus* | USNM | 317966 | https://n2t.net/ark:/87602/m4/M615 |
| *Hapalemur* | *occidentalis* | AMNH | 100823 | https://doi.org/10.17602/M2/M56603 |
| *Indri* | *Indri* | AMNH | 100503 | https://doi.org/10.17602/M2/M217 |
| *Indri* | *Indri* | AMNH | 100504 | https://doi.org/10.17602/M2/M225 |
| *Indri* | *indri* | AMNH | 100507 | https://n2t.net/ark:/87602/m4/M1945 |
| *Indri* | *Indri* | AMNH | 100508 | https://n2t.net/ark:/87602/m4/M1943 |
| *Indri* | *Indri* | AMNH | 185638 | https://doi.org/10.17602/M2/M221 |
| *Indri* | *Indri* | BMNH | 1981.72 | https://doi.org/10.17602/M2/M52984 |
| *Indri* | *Indri* | BMNH | 35.1.8.2 | https://doi.org/10.17602/M2/M52979 |
| *Indri* | *Indri* | USNM | 16197 | https://doi.org/10.17602/M2/M53044 |
| *Karanisia* | *clarki* | DPC | 21456K | https://n2t.net/ark:/87602/m4/M52896 |
| *Karanisia* | *clarki* | DPC | 21748B | https://n2t.net/ark:/87602/m4/M52889 |
| *Karanisia* | *clarki* | DPC | 21840H | https://n2t.net/ark:/87602/m4/M52892 |
| *Karanisia* | *clarki* | DPC | 22999F | https://n2t.net/ark:/87602/m4/M52954 |
| *Komba* | *minor* | KNM | SO438 | https://doi.org/10.17602/M2/M82699 |
| *Komba* | *robustus* | KNM | SO501 | https://doi.org/10.17602/M2/M82700 |
| *Lemur* | *catta* | AMNH | 100598 | https://doi.org/10.17602/M2/M233 |
| *Lemur* | *catta* | AMNH | 100821 | https://doi.org/10.17602/M2/M237 |
| *Lemur* | *catta* | AMNH | 170737 | https://doi.org/10.17602/M2/M229 |
| *Lemur* | *catta* | AMNH | 170740 | https://doi.org/10.17602/M2/M245 |
| *Lemur* | *catta* | AMNH | 170741 | https://doi.org/10.17602/M2/M249 |
| *Lemur* | *catta* | AMNH | 170743 | https://doi.org/10.17602/M2/M241 |
| *Lemur* | *catta* | AMNH | 201183 | https://doi.org/10.17602/M2/M56629 |
| *Lemur* | *catta* | DLC | 7142f | https://doi.org/10.17602/M2/M56636 |
| *Lemur* | *catta* | DPC | 92 | https://n2t.net/ark:/87602/m4/M56639 |
| *Lemur* | *catta* | USNM | 395515 | https://doi.org/10.17602/M2/M56755 |
| *Lemur* | *catta* | USNM | 589576 | https://doi.org/10.17602/M2/M52877 |
| *Lepilemur* | *edwardsi* | AMNH | 100623 | https://doi.org/10.17602/M2/M56601 |
| *Lepilemur* | *edwardsi* | AMNH | 100642 | https://doi.org/10.17602/M2/M253 |
| *Lepilemur* | *leucopus* | AMNH | 170557 | https://doi.org/10.17602/M2/M56614 |
| *Lepilemur* | *leucopus* | AMNH | 170559 | https://doi.org/10.17602/M2/M56615 |
| *Lepilemur* | *leucopus* | AMNH | 170561 | https://doi.org/10.17602/M2/M56616 |
| *Lepilemur* | *leucopus* | AMNH | 170564 | https://doi.org/10.17602/M2/M56617 |
| *Lepilemur* | *leucopus* | AMNH | 170568 | https://doi.org/10.17602/M2/M56618 |
| *Lepilemur* | *leucopus* | AMNH | 170569 | https://doi.org/10.17602/M2/M266 |
| *Lepilemur* | *leucopus* | AMNH | 170574 | https://doi.org/10.17602/M2/M56619 |
| *Lepilemur* | *leucopus* | AMNH | 170576 | https://doi.org/10.17602/M2/M257 |
| *Lepilemur* | *leucopus* | AMNH | 170578 | https://doi.org/10.17602/M2/M261 |
| *Lepilemur* | *leucopus* | DPC | 18839 | https://n2t.net/ark:/87602/m4/M56714 |
| *Lepilemur* | *leucopus* | DPC | 18901 | https://n2t.net/ark:/87602/m4/M56717 |
| *Lepilemur* | *leucopus* | DPC | 18951 | https://n2t.net/ark:/87602/m4/M56723 |
| *Lepilemur* | *microdon* | BMNH | 1981.762 | https://doi.org/10.17602/M2/M52640 |
| *Lepilemur* | *microdon* | BMNH | 1939.128 | https://doi.org/10.17602/M2/M52700 |
| *Lepilemur* | *mustelinus* | AMNH | 170790 | https://doi.org/10.17602/M2/M56623 |
| *Lepilemur* | *mustelinus* | AMNH | 170795 | https://doi.org/10.17602/M2/M56624 |
| *Lepilemur* | *mustelinus* | USNM | 49668 | https://doi.org/10.17602/M2/M56748 |
| *Lepilemur* | *ruficaudatus* | AMNH | 100612 | https://doi.org/10.17602/M2/M270 |
| *Lepilemur* | *ruficaudatus* | AMNH | 100622 | https://doi.org/10.17602/M2/M55842 |
| *Lepilemur* | *septentrionalis* | DPC | 12816 | https://n2t.net/ark:/87602/m4/M57693 |
| *Lepilemur* | *sp* | BMNH | 1897.12.2.2 | https://doi.org/10.17602/M2/M52684 |
| *Lepilemur* | sp | DPC | 13629 | https://n2t.net/ark:/87602/m4/M29587 |
| *Lepilemur* | *sp* | NHMB | QW 1606 | https://doi.org/10.17602/M2/M52710 |
| *Loris* | *tardigradus* | AMNH | 34258 | https://doi.org/10.17602/M2/M55773 |
| *Loris* | *tardigradus* | AMNH | 150062 | https://doi.org/10.17602/M2/M276 |
| *Loris* | *tardigradus* | AMNH | 165931 | https://doi.org/10.17602/M2/M273 |
| *Loris* | *tardigradus* | AMNH | 217303 | https://doi.org/10.17602/M2/M284 |
| *Loris* | *tardigradus* | DPC | 42 | https://n2t.net/ark:/87602/m4/M56638 |
| *Loris* | *tardigradus* | UAAC | 972.4.2 | https://doi.org/10.17602/M2/M56747 |
| *Loris* | *tardigradus* | UWBM | 59976 | https://doi.org/10.17602/M2/M52239 |
| *Megaladapis* | *edwardsi* | UA | 4621 | https://n2t.net/ark:/87602/m4/M82702 |
| *Megaladapis* | *sp* | DPC | 11787 | https://n2t.net/ark:/87602/m4/M56806 |
| *Megaladapis* | *sp* | DPC | 17218 | https://n2t.net/ark:/87602/m4/M56805 |
| *Megaladapis* | *sp* | DPC | 18935 | https://n2t.net/ark:/87602/m4/M56808 |
| *Megaladapis* | *sp* | DPC | NN | https://n2t.net/ark:/87602/m4/M56807 |
| *Mesopropithecus* | *dolichobranchion* | DPC | 9903 | https://doi.org/10.17602/M2/M82704 |
| *Mesopropithecus* | *pithecoides* | UA | 4848 | https://doi.org/10.17602/M2/M82710 |
| *Mesopropithecus* | *pithecoides* | UA | 4849 | https://doi.org/10.17602/M2/M82708 |
| *Microcebus* | *murinus* | AMNH | 100844 | https://doi.org/10.17602/M2/M56606 |
| *Microcebus* | *murinus* | AMNH | 100846 | https://doi.org/10.17602/M2/M56607 |
| *Microcebus* | *murinus* | AMNH | 174483 | https://doi.org/10.17602/M2/M308 |
| *Microcebus* | *murinus* | AMNH | 174489 | https://doi.org/10.17602/M2/M316 |
| *Microcebus* | *murinus* | AMNH | 174498 | https://doi.org/10.17602/M2/M303 |
| *Microcebus* | *murinus* | AMNH | 174530 | https://doi.org/10.17602/M2/M288 |
| *Microcebus* | *murinus* | AMNH | 174531 | https://doi.org/10.17602/M2/M312 |
| *Microcebus* | *murinus* | AMNH | 174533 | https://doi.org/10.17602/M2/M292 |
| *Microcebus* | *murinus* | AMNH | 174534 | https://doi.org/10.17602/M2/M296 |
| *Microcebus* | *murinus* | AMNH | 174537 | https://doi.org/10.17602/M2/M56627 |
| *Microcebus* | *murinus* | DLC | 7065 | https://n2t.net/ark:/87602/m4/M56653 |
| *Microcebus* | *murinus* | DLC | 893m | https://doi.org/10.17602/M2/M56633 |
| *Microcebus* | *murinus* | DLC | NN02 | https://doi.org/10.17602/M2/M56637 |
| *Microcebus* | *murinus* | DPC | 22078 | https://n2t.net/ark:/87602/m4/M56726 |
| *Microcebus* | *murinus* | DPC | 22093 | https://n2t.net/ark:/87602/m4/M56729 |
| *Microcebus* | *murinus* | MCZ | 44843 | https://doi.org/10.17602/M2/M56740 |
| *Microcebus* | *murinus* | MCZ | 45125 | https://doi.org/10.17602/M2/M56744 |
| *Microcebus* | *rufus* | AMNH | 100669 | https://doi.org/10.17602/M2/M56602 |
| *Microcebus* | *rufus* | AMNH | 174360 | https://doi.org/10.17602/M2/M56625 |
| *Microcebus* | *sambiranensis* | AMNH | 100663 | https://doi.org/10.17602/M2/M56599 |
| *Microcebus* | sp | DPC | 13653 | https://n2t.net/ark:/87602/m4/M56704 |
| *Microcebus* | sp | DPC | 13669 | https://n2t.net/ark:/87602/m4/M56707 |
| *Microcebus* | sp | DPC | 18902 | https://n2t.net/ark:/87602/m4/M56720 |
| *Microcebus* | *sp* | USNM | 545004 | https://doi.org/10.17602/M2/M56757 |
| *Mirza* | *coquereli* | AMNH | 100832 | https://doi.org/10.17602/M2/M320 |
| *Mirza* | *coquereli* | DPC | 137 | https://doi.org/10.17602/M2/M82737 |
| *Mirza* | *coquereli* | DPC | 1139 | https://doi.org/10.17602/M2/M82741 |
| *Mirza* | *coquereli* | MCZ | 45124 | https://n2t.net/ark:/87602/m4/M595 |
| *Mirza* | *zaza* | DPC | 2322f | https://doi.org/10.17602/M2/M56635 |
| *Nycticeboides* | *sp* | YGSP | 8091 | https://doi.org/10.17602/M2/M82759 |
| *Nycticebus* | *bengalensis* | AMNH | 87279 | https://doi.org/10.17602/M2/M372 |
| *Nycticebus* | *bengalensis* | AMNH | 164442 | https://doi.org/10.17602/M2/M332 |
| *Nycticebus* | *bengalensis* | AMNH | 183827 | https://doi.org/10.17602/M2/M328 |
| *Nycticebus* | *coucang* | AMNH | 106653 | https://doi.org/10.17602/M2/M339 |
| *Nycticebus* | *coucang* | SBU | PNL-01 | https://doi.org/10.17602/M2/M472 |
| *Nycticebus* | *coucang* | UAMZ | 4657 | https://doi.org/10.17602/M2/M51831 |
| *Nycticebus* | *javanicus* | AMNH | 101508 | https://doi.org/10.17602/M2/M342 |
| *Nycticebus* | *javanicus* | AMNH | 101782 | https://doi.org/10.17602/M2/M345 |
| *Nycticebus* | *javanicus* | AMNH | 102845 | https://doi.org/10.17602/M2/M347 |
| *Nycticebus* | *pygmaeus* | BMNH | 1928.7.1.12 | https://doi.org/10.17602/M2/M51804 |
| *Nycticebus* | *pygmaeus* | MCZ | 36035 | https://doi.org/10.17602/M2/M51815 |
| *Otolemur* | *crassicaudatus* | AMNH | 88060 | https://doi.org/10.17602/M2/M51030 |
| *Otolemur* | *crassicaudatus* | AMNH | 88061 | https://doi.org/10.17602/M2/M51039 |
| *Otolemur* | *crassicaudatus* | AMNH | 88062 | https://doi.org/10.17602/M2/M51048 |
| *Otolemur* | *crassicaudatus* | AMNH | 216240 | https://doi.org/10.17602/M2/M51722 |
| *Otolemur* | *crassicaudatus* | AMNH | 216244 | https://doi.org/10.17602/M2/M51729 |
| *Pachylemur* | *jullyi* | UA | 5188 | https://doi.org/10.17602/M2/M82764 |
| *Pachylemur* | *jullyi* | UA | 5201 | https://doi.org/10.17602/M2/M82765 |
| *Palaeopropithecus* | *kelyus* | UA | 6191 | https://doi.org/10.17602/M2/M83022 |
| *Palaeopropithecus* | *maximus* | UA | 196 | https://doi.org/10.17602/M2/M83023 |
| *Palaeopropithecus* | *maximus* | UA | 4453 | https://doi.org/10.17602/M2/M83024 |
| *Palaeopropithecus* | *sp* | DPC | 13708 | https://n2t.net/ark:/87602/m4/M56710 |
| *Perodicticus* | *potto* | AMNH | 31252 | https://doi.org/10.17602/M2/M368 |
| *Perodicticus* | *potto* | AMNH | 239437 | https://doi.org/10.17602/M2/M56631 |
| *Perodicticus* | *potto* | AMNH | 241117 | https://doi.org/10.17602/M2/M364 |
| *Perodicticus* | *potto* | AMNH | 269851 | https://doi.org/10.17602/M2/M360 |
| *Perodicticus* | *potto* | AMNH | 269860 | https://doi.org/10.17602/M2/M352 |
| *Perodicticus* | *potto* | AMNH | 269907 | https://doi.org/10.17602/M2/M349 |
| *Perodicticus* | *potto* | DPC | 175 | https://n2t.net/ark:/87602/m4/M56640 |
| *Perodicticus* | *potto* | USNM | 481739 | https://doi.org/10.17602/M2/M56756 |
| *Perodicticus* | *potto* | UWBM | 39440 | https://doi.org/10.17602/M2/M51007 |
| *Perodicticus* | *potto ibeanus* | AMNH | 52592 | https://doi.org/10.17602/M2/M83025 |
| *Perodicticus* | *potto ibeanus* | AMNH | 119066 | https://doi.org/10.17602/M2/M56608 |
| *Phaner* | *furcifer* | AMNH | 100829 | https://doi.org/10.17602/M2/M376 |
| *Phaner* | *furcifer* | MCZ | 44953 | https://n2t.net/ark:/87602/m4/M591 |
| *Plesiopithecus* | *teras* | DPC | 11636 | https://n2t.net/ark:/87602/m4/M4341 |
| *Plesiopithecus* | *teras* | DPC | 13607 | https://n2t.net/ark:/87602/m4/M55714 |
| *Prolemur* | *simus* | BMNH | 1984.10.20.4 | https://doi.org/10.17602/M2/M83026 |
| *Prolemur* | *simus* | DPC | 7845 | https://doi.org/10.17602/M2/M50755 |
| *Prolemur* | *simus* | DPC | 7870 | https://n2t.net/ark:/87602/m4/M50762 |
| *Prolemur* | *simus* | DPC | 7907 | https://n2t.net/ark:/87602/m4/M50830 |
| *Prolemur* | *simus* | DPC | 7917 | https://n2t.net/ark:/87602/m4/M50861 |
| *Prolemur* | *simus* | DPC | 7932 | https://n2t.net/ark:/87602/m4/M50774 |
| *Prolemur* | *simus* | DPC | 7955 | https://n2t.net/ark:/87602/m4/M50873 |
| *Prolemur* | *simus* | DPC | 7964 | https://n2t.net/ark:/87602/m4/M50780 |
| *Prolemur* | *simus* | DPC | 12765 | https://n2t.net/ark:/87602/m4/M50806 |
| *Prolemur* | *simus* | DPC | 12767 | https://n2t.net/ark:/87602/m4/M50812 |
| *Prolemur* | *simus* | DPC | 12768 | https://n2t.net/ark:/87602/m4/M50821 |
| *Prolemur* | *simus* | DPC | 10936B | https://n2t.net/ark:/87602/m4/M50797 |
| *Prolemur* | *simus* | DPC | 10936A | https://n2t.net/ark:/87602/m4/M50791 |
| *Pronycticebus* | *gaudryi* | MNHN | 11056 | https://doi.org/10.17602/M2/M83027 |
| *Propithecus* | *coronatus* | AMNH | 17356 | https://doi.org/10.17602/M2/M388 |
| *Propithecus* | *diadema* | AMNH | 100633 | https://doi.org/10.17602/M2/M384 |
| *Propithecus* | *diadema* | USNM | 63347 | https://doi.org/10.17602/M2/M50679 |
| *Propithecus* | *diadema* | USNM | 63349 | https://doi.org/10.17602/M2/M391 |
| *Propithecus* | *diadema* | USNM | 63350 | https://doi.org/10.17602/M2/M56749 |
| *Propithecus* | *diadema* | USNM | 84385 | https://doi.org/10.17602/M2/M50713 |
| *Propithecus* | *edwardsi* | USNM | a21171 | https://doi.org/10.17602/M2/M50670 |
| *Propithecus* | *edwardsi* | USNM | 63351 | https://doi.org/10.17602/M2/M56750 |
| *Propithecus* | *sp* | USNM | 63348 | https://doi.org/10.17602/M2/M83031 |
| *Propithecus* | *verreauxi* | AMNH | 16699 | https://doi.org/10.17602/M2/M400 |
| *Propithecus* | *verreauxi* | AMNH | 100827 | https://doi.org/10.17602/M2/M404 |
| *Propithecus* | *verreauxi* | AMNH | 170463 | https://doi.org/10.17602/M2/M56610 |
| *Propithecus* | *verreauxi* | AMNH | 170467 | https://doi.org/10.17602/M2/M56611 |
| *Propithecus* | *verreauxi* | AMNH | 170470 | https://doi.org/10.17602/M2/M56612 |
| *Propithecus* | *verreauxi* | AMNH | 170473 | https://doi.org/10.17602/M2/M56613 |
| *Propithecus* | *verreauxi* | USNM | 257397 | https://doi.org/10.17602/M2/M396 |
| *Propithecus* | *sp* | DPC | 3732 | https://n2t.net/ark:/87602/m4/M56643 |
| *Propithecus* | *sp* | DPC | 3799 | https://n2t.net/ark:/87602/m4/M56649 |
| *Propithecus* | *sp* | DPC | 13625 | https://n2t.net/ark:/87602/m4/M56698 |
| *Propotto* | *leakeyi* | KMN-CA | 2195 | https://doi.org/10.17602/M2/M55715 |
| *Propotto* | *leakeyi* | KMN-KO | 101 | https://doi.org/10.17602/M2/M55716 |
| *Propotto* | *leakeyi* | KMN-SO | 508 | https://doi.org/10.17602/M2/M55717 |
| *Teilhardina* | *sp* | IRSNB | 455 | https://doi.org/10.17602/M2/M83342 |
| *Teilhardina* | *sp* | IRSNB | 457 | https://doi.org/10.17602/M2/M83335 |
| *Teilhardina* | *sp* | IRSNB | 4291 | https://doi.org/10.17602/M2/M83348 |
| *Teilhardina* | *sp* | IRSNB | 65 | https://doi.org/10.17602/M2/M83351 |
| *Varecia* | *rubra* | AMNH | 100513 | https://doi.org/10.17602/M2/M49550 |
| *Varecia* | *rubra* | AMNH | 100514 | https://doi.org/10.17602/M2/M444 |
| *Varecia* | *variegata* | AMNH | 17338 | https://doi.org/10.17602/M2/M460 |
| *Varecia* | *variegata* | AMNH | 18041 | https://doi.org/10.17602/M2/M456 |
| *Varecia* | *variegata* | AMNH | 100512 | https://doi.org/10.17602/M2/M56758 |
| *Varecia* | *variegata* | AMNH | 245092 | https://doi.org/10.17602/M2/M452 |
| *Varecia* | *variegata* | USNM | 84381 | https://doi.org/10.17602/M2/M56752 |
| *Varecia* | *variegata* | USNM | 84383 | https://doi.org/10.17602/M2/M56753 |
| *Wadilemur* | *elegans* | DPC | 13439 | https://n2t.net/ark:/87602/m4/M49543 |
| *Wadilemur* | *elegans* | DPC | 16872 | https://n2t.net/ark:/87602/m4/M56711 |

Table S2: Dietary classifications with primary references

| Genus | Dietary category | References |
| --- | --- | --- |
| *Arctocebus* | Insectivory | [4,5] |
| *Avahi* | Folivory | [6–8] |
| *Cheirogaleus* | Frugivory | [8–10] |
| *Eulemur* | Frugivory | [11–14] |
| *Euoticus* | Frugivory | [5] |
| *Galago* | Insectivory | [15–17] |
| *Galagoides* | Insectivory | [4,5,18] |
| *Hapalemur* | Folivory | [19] |
| *Indri* | Folivory | [20,21] |
| *Lemur* | Frugivory | [22,23] |
| *Lepilemur* | Folivory | [24] |
| *Loris* | Insectivory | [25,26] |
| *Microcebus* | Insectivory | [8,9,27] |
| *Mirza* | Frugivory | [9] |
| *Nycticebus* | Frugivory | [28–31] |
| *Otolemur* | Frugivory | [18,32] |
| *Perodicticus* | Frugivory | [4,5] |
| *Phaner* | Frugivory | [9,33] |
| *Prolemur* | Folivory | [34] |
| *Propithecus* | Folivory | [13,14,35,36] |
| *Sciurocheirus* (= *Galago alleni*) | Frugivory | [4,5] |
| *Varecia* | Frugivory | [14,37,38] |

Table S3: Support from information criteria for alternate models of the evolution of each dental topography metric over the complete lemur clade and the clade originating during the Oligocene (excluding Chiromyiformes and Megaladapis).

| Clade |  | BM AICc | BM Weight | EB AICc | EB Weight | OU AICc | OU Weight |
| --- | --- | --- | --- | --- | --- | --- | --- |
| Lemurs | DNE | -152.9 | 0.65 | -149.3 | 0.17 | -149.3 | 0.18 |
| Lemurs | DNE CV | -18.2 | 0.069 | -15.6 | 0.018 | -23.4 | 0.91 |
| Lemurs | RFI | -105 | 0.64 | -102.4 | 0.17 | -102.6 | 0.19 |
| Lemurs (without fossil taxa) | DNE | -137 | 0.65 | -134.4 | 0.18 | -134.3 | 0.17 |
| Lemurs (without fossil taxa) | DNE CV | -14.7 | 0.10 | -12 | 0.03 | -19 | 0.87 |
| Lemurs (without fossil taxa) | RFI | -93.2 | 0.62 | -90.4 | 0.16 | -91.1 | 0.22 |
| Oligocene lemurs | DNE | -129.4 | 0.49 | -126.5 | 0.12 | -128.9 | 0.40 |
| Oligocene lemurs | DNE CV | -13.2 | 0.30 | -10.4 | 0.07 | -14.6 | 0.62 |
| Oligocene lemurs | RFI | -85.9 | 0.62 | -83 | 0.15 | -83.9 | 0.23 |

Table S4: Model coefficients from multilevel models comparing rates of evolution during the Eocene and Oligocene to rates of evolution outside of these intervals. Negative coefficients suggest lower rates of evolution during the hypothesized interval; positive coefficients suggest higher rates of evolution.

|  | Eocene rates coefficient | Eocene rates 95% CI | Oligocene rates coefficient | Oligocene rates 95% CI |
| --- | --- | --- | --- | --- |
| MR model | -0.18 | -0.62 : 0.26 | 0.64 | 0.37 : 0.92 |
| PDR model | -0.29 | -0.47: -0.11 | 0.19 | 0 : 38 |

1. Spradley JP, Pampush JD, Morse PE, Kay RF. 2017 Smooth operator: the effects of different 3D mesh retriangulation protocols on the computation of Dirichlet normal energy. *American journal of physical anthropology* **163**, 94–109.

2. Bürkner P-C. 2017 brms: An R package for Bayesian multilevel models using Stan. *Journal of Statistical Software* **80**, 1–28.

3. Szalay FS, Delson E. 1979 *Evolutionary history of the primates*. Academic Press.

4. Charles-Dominique P. 1977 *Ecology and behaviour of nocturnal primates: prosimians of equatorial West Africa*. Columbia University Press.

5. Charles-Dominique P. 1979 Field studies of lorisid behavior: methodological aspects. *The study of prosimian behavior*

6. Ganzhorn JU, Abraham JP, Razanahoera-Rakotomalala M. 1985 Some aspects of the natural history and food selection ofAvahi laniger. *Primates* **26**, 452–463.

7. Harcourt C. 1991 Diet and behaviour of a nocturnal lemur, Avahi laniger, in the wild. *Journal of Zoology* **223**, 667–674.

8. Lahann P. 2007 Feeding ecology and seed dispersal of sympatric cheirogaleid lemurs (Microcebus murinus, Cheirogaleus medius, Cheirogaleus major) in the littoral rainforest of south‐east Madagascar. *Journal of Zoology* **271**, 88–98.

9. Hladik CM, Charles-Dominique P, Petter J-J. 1980 Feeding strategies of five nocturnal prosimians in the dry forest of the west coast of Madagascar. *Nocturnal Malagasy primates: ecology, physiology, and behavior* , 41–73.

10. Fietz J, Ganzhorn JU. 1999 Feeding ecology of the hibernating primate Cheirogaleus medius: how does it get so fat? *Oecologia* **121**, 157–164.

11. Overdorff DJ. 1992 Differential patterns in flower feeding by Eulemur fulvus rufus and Eulemur rubriventer in Madagascar. *American Journal of Primatology* **28**, 191–203.

12. Vasey N. 2002 Niche separation in Varecia variegata rubra and Eulemur fulvus albifrons: II. Intraspecific patterns. *American Journal of Physical Anthropology* **118**, 169–183.

13. Sato H, Santini L, Patel ER, Campera M, Yamashita N, Colquhoun IC, Donati G. 2016 Dietary flexibility and feeding strategies of Eulemur: a comparison with Propithecus. *International Journal of Primatology* **37**, 109–129.

14. Erhart EM, Tecot SR, Grassi C. 2018 Interannual Variation in Diet, Dietary Diversity, and Dietary Overlap in Three Sympatric Strepsirrhine Species in Southeastern Madagascar. *International Journal of Primatology* **39**, 289–311.

15. Harcourt C. 1986 Seasonal variation in the diet of South African galagos. *International Journal of Primatology* **7**, 491–506.

16. Bearder SK, Martin RD. 1980 Acacia gum and its use by bushbabies, Galago senegalensis (Primates: Lorisidae). *International Journal of Primatology* **1**, 103–128.

17. Burrows AM, Nash LT. 2010 Searching for dental signals of exudativory in Galagos. In *The evolution of exudativory in primates*, pp. 211–233. Springer.

18. Harcourt CS, Nash LT. 1986 Species differences in substrate use and diet between sympatric galagos in two Kenyan coastal forests. *Primates* **27**, 41–52.

19. Overdorff DJ, Strait SG, Telo A. 1997 Seasonal variation in activity and diet in a small‐bodied folivorous primate, Hapalemur griseus, in southeastern Madagascar. *American Journal of Primatology* **43**, 211–223.

20. Britt A, Randriamandratonirina NJ, Glasscock KD, Iambana BR. 2002 Diet and feeding behaviour of Indri indri in a low-altitude rain forest. *Folia Primatologica* **73**, 225–239.

21. Powzyk JA, Mowry CB. 2003 Dietary and feeding differences between sympatric Propithecus diadema diadema and Indri indri. *International Journal of Primatology* **24**, 1143–1162.

22. Hladik CM. 1979 Diet and ecology of prosimians. *The study of prosimian behavior* , 307–357.

23. Gould L. 2006 Lemur catta ecology: what we know and what we need to know. In *Lemurs*, pp. 255–274. Springer.

24. Thalmann U. 2001 Food resource characteristics in two nocturnal lemurs with different social behavior: Avahi occidentalis and Lepilemur edwardsi. *International Journal of Primatology* **22**, 287–324.

25. Nekaris KAI, Rasmussen DT. 2003 Diet and feeding behavior of Mysore slender lorises. *International Journal of Primatology* **24**, 33–46.

26. Nekaris KAI. 2005 Foraging behaviour of the slender loris (Loris lydekkerianus lydekkerianus): implications for theories of primate origins. *Journal of Human Evolution* **49**, 289–300.

27. Dammhahn M, Kappeler PM. 2008 Small-scale coexistence of two mouse lemur species (Microcebus berthae and M. murinus) within a homogeneous competitive environment. *Oecologia* **157**, 473–483.

28. Streicher U. 2004 Aspects of the ecology and conservation of the pygmy loris Nycticebus pygmaeus in Vietnam. Dissertation. Germany: Ludwig-Maximilians Universität.

29. Streicher U. 2009 Diet and feeding behaviour of pygmy lorises (Nycticebus pygmaeus) in Vietnam. *Vietnamese Journal of Primatology* **3**, 37–44.

30. Wiens F, Zitzmann A, Hussein NA. 2006 Fast food for slow lorises: is low metabolism related to secondary compounds in high-energy plant diet? *Journal of Mammalogy* **87**, 790–798.

31. Rode-Margono EJ, Nijman V, Wirdateti NK, Nekaris KAI. 2014 Ethology of the critically endangered Javan slow loris Nycticebus javanicus E. Geoffroy Saint-Hilaire in West Java. *Asian Primates* **4**, 27–41.

32. Masters JC, Lumsden WHR, Young DA. 1988 Reproductive and dietary parameters in wild greater galago populations. *International Journal of Primatology* **9**, 573.

33. Nash LT. 1986 Dietary, behavioral, and morphological aspects of gummivory in primates. *American journal of physical anthropology* **29**, 113–137.

34. Olson ER, Marsh RA, Bovard BN, Randrianarimanana HL, Ravaloharimanitra M, Ratsimbazafy JH, King T. 2013 Habitat preferences of the Critically Endangered Greater bamboo lemur (Prolemur simus) and densities of one of its primary food sources, Madagascar giant bamboo (Cathariostachys madagascariensis), in sites with different degrees of anthropogenic and natural disturbance. *International Journal of Primatology* **34**, 486–499.

35. Hemingway CA. 1996 Morphology and phenology of seeds and whole fruit eaten by Milne-Edwards’ sifaka, Propithecus diadema edwardsi, in Ranomafana National Park, Madagascar. *International journal of primatology* **17**, 637–659.

36. Norscia I, Carrai V, Borgognini-Tarli SM. 2006 Influence of dry season and food quality and quantity on behavior and feeding strategy of Propithecus verreauxi in Kirindy, Madagascar. *International Journal of Primatology* **27**, 1001–1022.

37. Balko EA. 1998 A behaviorally plastic response to forest composition and logging disturbance by Varecia variegata in Ranomafana National Park, Madagascar. *PhD dissertation, Syracuse University*

38. Vasey N. 2000 Niche separation in Varecia variegata rubra and Eulemur fulvus albifrons: I. Interspecific patterns. *American Journal of Physical Anthropology* **112**, 411–431.
